# Whole genome sequencing reveals within-host genetic changes in paired meningococcal carriage isolates from Ethiopia

**DOI:** 10.1186/s12864-017-3806-3

**Published:** 2017-05-25

**Authors:** Guro K. Bårnes, Ola Brønstad Brynildsrud, Bente Børud, Bereket Workalemahu, Paul A. Kristiansen, Demissew Beyene, Abraham Aseffa, Dominique A. Caugant

**Affiliations:** 1 Division for Infection Control and Environmental Health, Norwegian Institute of Public Health, Oslo, Norway; 2 WHO Collaborating Center for Reference and Research on Meningococci, Norwegian Institute of Public Health, Oslo, Norway; 3 Faculty of Medicine, University of Oslo, Oslo, Norway; 4 Arba Minch College of Health Sciences, Arba Minch, Ethiopia; 5 Armauer Hansen Research Institute, Addis Ababa, Ethiopia; 6 Hamlin Fistula Ethiopia, Addis Ababa, Ethiopia

**Keywords:** *Neisseria meningitidis*, Carriage, Ethiopia, Meningitis belt, Whole genome sequencing, Core genome, Genetics

## Abstract

**Background:**

Meningococcal colonization is a prerequisite for transmission and disease, but the bacterium only very infrequently causes disease while asymptomatic carriage is common. Carriage is highly dynamic, showing a great variety across time and space within and across populations, but also within individuals. The understanding of genetic changes in the meningococcus during carriage, when the bacteria resides in its natural niche, is important for understanding not only the carriage state, but the dynamics of the entire meningococcal population.

**Results:**

Paired meningococcal isolates, obtained from 50 asymptomatic carriers about 2 months apart were analyzed with whole genome sequencing (WGS). Phylogenetic analysis revealed that most paired isolates from the same individual were closely related, and the average and median number of allelic differences between paired isolates defined as the same strain was 35. About twice as many differences were seen between isolates from different individuals within the same sequence type (ST). In 8%, different strains were detected at different time points. A difference in ST was observed in 6%, including an individual who was found to carry three different STs over the course of 9 weeks. One individual carried different strains from the same ST.

In total, 566 of 1605 cgMLST genes had undergone within-host genetic changes in one or more pairs. The most frequently changed cgMLST gene was *relA* that was changed in 47% of pairs. Across the whole genome, *pilE*, differed mostly, in 85% of the pairs. The most frequent mechanisms of genetic difference between paired isolates were phase variation and recombination, including gene conversion. Different STs showed variation with regard to which genes that were most frequently changed, mostly due to absence/presence of phase variation.

**Conclusions:**

This study revealed within-host genetic differences in meningococcal isolates during short-term asymptomatic carriage. The most frequently changed genes were genes belonging to the pilin family, the restriction/modification system, opacity proteins and genes involved in glycosylation. Higher resolution genome-wide sequence typing is necessary to resolve the diversity of isolates and reveals genetic differences not discovered by traditional typing schemes, and would be the preferred choice of technology.

**Electronic supplementary material:**

The online version of this article (doi:10.1186/s12864-017-3806-3) contains supplementary material, which is available to authorized users.

## Background

*Neisseria meningitidis*, a Gram-negative diplococcus found only in humans, colonizes the oropharynx and normally resides there without causing disease. The carriage state of meningococci has been known for a long time and the first carriage studies were done already in the 1890s [1]. Carriage prevalence varies geographically, but is usually around 5–10% [2, 3]. Colonization of the host mucosa is a prerequisite for transmission to other individuals, mainly through close contact and airborne droplets. Rarely, colonization progresses to invasive disease, usually shortly after acquisition of the bacteria [4, 5]. If the bacteria reach the bloodstream or the cerebrospinal fluid, they can cause meningitis and/or septicemia [6, 7].

Meningococci are classified into 12 different serogroups based on their polysaccharide capsule [8]. Invasive disease is most often caused by serogroups A, B, C, W, X and Y meningococci, although non-capsulated bacteria, which are the most commonly found meningococci among carriers [9], have also been reported to cause disease [10–12]. The presence of a capsule is an advantage in invasive disease, helping the bacteria evade complement-mediated and phagocytic killing by the host in the bloodstream [13], while the loss of capsule seems to enhance the bacteria’s ability for colonization [14, 15].

Meningococcal disease occurs endemically throughout the world, and all continents have seen epidemics, but no region more than the sub-Saharan meningitis belt. During epidemics, carriage prevalence of the outbreak strain can increase many fold [16] and these epidemic incidences are related more to the substantial increase in transmission and colonization, and less to changes in the ratio of cases to carriers [17]. Crowding, within households [18] or in settings like military camps and university campuses [9, 19], increases the likelihood of carriage, as do other risk factors such as smoking [20] and respiratory tract infections [21]. Most cases of disease are sporadic and patients rarely have been in contact with another patient [9], thus, transmission by healthy carriers is a necessity for effectively spreading disease. Therefore, vaccines that prevent colonization are important in controlling disease prevalence. Conjugated vaccines that target the polysaccharide capsule of the meningococcus have been shown to be highly effective in doing so, inducing herd protection in addition to protecting vaccinated individuals against invasive disease [22]. Carrier studies are therefore needed to support and guide the introduction of meningococcal conjugate vaccines, to understand transmission patterns of different strains and to measure within-host evolutionary changes and adaptation.

The majority of meningococcal carriage studies have been cross-sectional studies, providing a snapshot of the prevalence and diversity at a given time. Such studies do not give any information on the duration of carriage or the genetic changes that occur in the bacterial population within the host during colonization. A few longitudinal carriage studies have been done following individuals with multiple samples over time. A study from Czech Republic, Greece and Norway showed that 43–69% of the individuals found to be carriers were still carriers after 5–6 months [9] and a study in the UK showed that only 44.1% of students identified as carriers were still carriers after 1–2 months [23]. A study in Belgian schoolchildren in the 1980s showed a mean duration of carriage estimated to 11.7 months [24], whereas a recent study from Africa found the average carriage duration to be considerably shorter, at 3.4 months [25]. However, among the British students who cleared carriage within the first months, 19% were recolonized at 6 months, and only 63.2% of the students were carriers of the same serogroup after 6 months [23]. This shows that the carriage state is not a steady-state, but is highly dynamic.

To characterize and differentiate the population of *N. meningitidis,* a variety of serological and molecular classification methods have been used. Multilocus sequence typing (MLST) which assigns isolates to a sequence type (ST), and eventually to a clonal complex, based on allelic variation in seven housekeeping genes, has been the most widely used method to study population structure in meningococci. Recent new technologies have allowed for rapid and affordable sequencing and comparison of larger parts of, or the whole bacterial genome. Whole genome sequencing (WGS) is providing us, in addition to higher resolution to distinguish among different strains, information on variation in genes that affect other functions of the bacteria, such as antibiotic susceptibility, virulence and adaptation to the host. For comparison and phylogenetic analyses it is suitable to use the core genome, comprising the genes that are present in all members of a population. In addition, in a diverse population like the meningococcus, the accessory genome that comprises variably present genes in isolates, is a substantial part of the genome and contributes to understanding population diversity. WGS has been proven helpful in tracing transmission and in guiding infection control responses [26]. For comparison between laboratories, defined core genome genes, like the core genome MLST (cgMLST) at the pubMLST database, have been used [27]. Although most widely applied in research, epidemiology and reference laboratories to date, with the continuous improvement of technology and development of bioinformatics knowledge, WGS is likely to become a part of clinical routine microbiology in the foreseeable future.

The meningococcus has multiple unique properties and mechanisms that favor genetic change and antigenic variation [28]. Repeat elements and DNA uptake sequences enhance acquisition of DNA from the environment [29, 30]. Recombination is by far the main evolutionary mechanism of antigenic diversity in the meningococcus [31, 32]. Based on MLST genes, the rate of recombination events has been estimated to be about 6.2–16.8 times higher than the rate of nucleotide mutation events [32], and observational studies have shown that a particular nucleotide site is at least 80 times more likely to change by recombination than by mutation [31]. Antigenic variation arises not only through permanent changes in the DNA, but also through a reversible phase variation mechanism [33], in regions with homopolymers or repeating short nucleotide sequences.

The knowledge of what kind of genetic changes that occurs in the meningococcus in vivo can help our understanding of the dynamics of the meningococcal genome. The investigation of within-host genetic changes during carriage can increase our understanding not only of the carriage state, but also the role of carriage in disease transmission and the spread of genetic traits in the meningococcal population. The genomic changes selected for in the oropharynx during interaction with the host might affect the virulence of the bacterium. Multiple studies have used WGS for investigations of *N. meningitidis* isolates over the last years, but to our knowledge no study has investigated the specific genetic changes occurring in meningococcal isolates from the same individuals during carriage. To identify the genetic changes occurring in meningococci during colonization of the oropharynx, the bacteria’s natural niche, meningococcal isolates obtained from the same individuals approximately two months apart were characterized by WGS.

## Methods

### Isolate collection

The meningococcal isolates analyzed were selected from a carriage study among healthy 1–29 year olds in the Arba Minch area in southern Ethiopia in 2014 [34], where a subgroup of individuals identified as asymptomatic carriers were followed by repeated oropharyngeal samples weekly over a period of 9 weeks. Samples were obtained by swabbing and direct plating onto selective agar plates in the field, before being transported in CO_2_-enriched containers to the laboratory within 6 h, for incubation at 37 °C overnight. Identification of *N. meningitidis* was made by examination of colony morphology and enzymatic testing [35]. The meningococcal isolates were stored in Greaves solution [36] at −80 °C, and transported on dry ice for further characterization at the Norwegian Institute of Public Health (NIPH).

Half of the individuals were vaccinated with either a monovalent serogroup A conjugate vaccine (MenAfriVac) or a tetravalent serogroup A + C + W + Y conjugate vaccine (Menveo) at the first sampling point. Two (nos. 4 and 16) of five carriers of serogroup W were vaccinated with the tetravalent vaccine containing serogroup W polysaccharide, whereas no other individuals were vaccinated with vaccines containing the serogroup which they were carrying. The individuals were asymptomatic and healthy throughout the study period and none reported to have taken any antibiotics during or 30 days prior to the study.

Individuals with paired isolates obtained at least 6 weeks apart were included (*n* = 50) and both the first and the last isolates were submitted for WGS analysis. For individuals where a strain replacement had occurred, MLST analysis was performed according to the method on the website (http://pubmlst.org/neisseria/) [37] for all available isolates taken during the 9 weeks follow up.

### Genome sequencing

Cultures were incubated overnight on blood agar plates at 37 °C in an atmosphere of 5% CO_2_. DNA was extracted using an automated MagNAPure isolation station and MagNAPure 96 DNA and Viral NA Small Volume Kit (Roche, Basel, Switzerland), according to the manufacturer's instructions. DNA quantity was assessed using the Qubit device (Invitrogen, Thermo Fisher Scientific Inc, Waltham, MA, US). For each isolate ≥50 ng of DNA was used for preparing the sequencing libraries with KAPA HyperPlus Kit (KAPA Biosystems, Wilmington, MA, US), following the KR1145 – v3.16 instructions from the manufacturer, with size selection of fragments ≤ 450 bp. Sequencing was performed on the Illumina MiSeq platform with MiSeq Reagent Kits v2 500-cycles (Illumina Inc., San Diego, CA, US) with 250 bp paired-end run modes. Fastq reads were trimmed and filtered using the software Trimmomatic v0.36 [38] with the KAPA adapters added to the filtering library, using the following settings: 2:30:10 for seed mismatches:palindrome clip threshold:simple clip threshold, minimum quality of 3 to keep a base used for leading and trailing end trimming, 3:15 for window size in bp and minimum quality, and a minimum trimmed length of 36 to keep reads. Sequence data were assembled *de novo* using the software SPAdes v3.8.0 [39] with default options as well as the --careful flag, using k-mer sizes 77, 99 and 127. Taxonomic labels were assigned using Kraken 0.10.5-beta [40]. Contigs shorter than 500 bp and contigs with an average kmer-coverage ≤ 5 were additionally filtered out using an in-house script. The final assembly files consisted of a median number of 71 contigs/sample (range: 46 to 121 contigs) with an average length of 29 503 bp, covering the ∼ 2.2 Mb of the *N. meningitidis* genome. The estimated average coverage varied between sequencing runs and ranged from 58.6 to 88.3.

### Genome comparison and phylogenetic analyses

Genomes were uploaded to the PubMLST.org database (http://pubmlst.org/neisseria/) (Additional file 1: Table S1), which is served by the Bacterial Isolate Genome Sequence Database (BIGSdb) platform [41]. For core genome analysis, the 1605 loci defined as the core genome in the database (*N. meningitidis* cgMLST v1.0) were used [42]. Incomplete loci were removed from individual pairs prior to calculations. For allelic comparisons, gene-by-gene analyses were performed using the Genome Comparator tool embedded within the PubMLST website. Analyzed genes were based on the annotated meningococcal genes available in the PubMLST database as of 27 Oct 2016. Distance matrices based on the allelic differences were created using the Neighbor-net method [43] and were used to visualize the phylogenetic network between all 100 isolates from the 50 individuals using SplitsTree4 v4.14.4) [44]. The same approach was used to create ST-specific split graphs for ST-11, ST-53, ST-192 and ST-2880. Within these four STs Bayesian analysis of population structure (BAPS) analyses were done using the program BAPS v6.0 [45], to formally define clusters (see Additional file 2: Figure S1). Allele numbers were used directly, and ambiguous, truncated and new alleles were set as missing data. For this analysis, the “Clustering of Individuals” module was used, using a maximum number of clusters from 1 to 10. The determined number of clusters was defined by a posterior probability > 0.975.

For phylogenetic analysis within each ST, a core genome single nucleotide polymorphism (SNP)-based approached was used. Briefly, within each ST the core genome mutations were inferred by ParSnp v1.2 [46], using the best assembly (as measured using contig N50) as reference. Putatively recombined regions were excluded using Gubbins v2.2.0 [47] with default options (raxml tree builder, GTRCAT model, max 5 iterations, filter taxa > 25% gaps, min 3 SNPs to define a recombination block, window size 100-10,000). Phylogenetic trees were created with RAxML v8.2.9 [48] under the GTRCAT model, using 1000 bootstrap replicates. The final bootstrapped SNP trees were annotated in Interactive Tree of Life [49].

For determination of mechanism of differences between paired isolates, sequences were uploaded and aligned in MEGA7 [50], and compared manually. Mechanisms were assigned as point mutation if only a single nucleotide difference was present in a window of 150 nucleotides, recombination if multiple nucleotide differences were present in the same area, phase variation if varying length of repeat nucleotides, and deletion or insertion were determined in relation to the majority of isolates. As Illumina sequencing is known to produce errors after long homopolymer tracts [51], only mononucleotide repeat sequences < 20 bases were included in the calculations. In cases where alleles were incomplete or missing in BIGSdb, missed gene sequences were to some extent retrieved when performing BLAST searches [52]. If incomplete sequences were retrieved via BLAST and genetic differences were found in pairs, they were included in the analysis of mechanisms.

## Results

### Study population and samples

The paired isolates from the same individuals were obtained between 6 and 9 weeks apart, with mean and median time periods of 8 and 9 weeks, respectively. The paired isolates are numbered 1–50, combined with A and B that indicate the two different time points. The samples were obtained from an equal distribution of male (52%) and female (48%) participants aged 2 to 29 years (Additional file 1: Table S1).

### Serogroups and sequence types

The majority of the 100 isolates were non-groupable (*n* = 73) and the remaining isolates belonged to serogroup Y (*n* = 15), W (*n* = 10) and X (*n* = 2). A total of 10 different STs were identified, with the majority of isolates belonging to ST-192 (*n* = 48) (Table 1). The initial isolate from each carrier was previously analyzed by MLST using Sanger sequencing [34], and the results found using WGS were identical with regards to ST assignment. WGS analysis of paired isolates revealed that 47 of the 50 individuals carried the same ST throughout the study period, whereas 3 out of 50 individuals had a change in ST over the course of two months (Table 1). In a fourth individual (no. 49), the ST remained the same, but nevertheless WGS revealed a change of strain (Table 1).

**Table 1:** Serogroups and sequence types of the study isolates

Serogroup	ST	No. of isolates	No. of individuals carrying the same strain at both time points	Isolate ID from individuals changing strains ^c^
NG (*cnl* ^a^)	53	11	4	48A, 49A, 49B
	192	48	23	47A, 50B
	198	5	2	47B
	11595	1	0	50A
	11597	2	1	
NG (not *cnl* ^b^)	35	2	1	
	175	4	2	
W	11	10	5	
X	11372	2	1	
Y	2880	15	7	48B
Total		100	46 individuals	4 individuals

*ST* Sequence type, *NG* non-groupable, *cnl* capsule null

^a^isolates harboring the capsule null locus, lacking the possibility to express capsule

^b^isolates not harboring the capsule null locus, but not harboring all genes necessary for capsule synthesis

^c^The number identifies the individual and the letters A and B, the first and the last isolates, respectively

### Allelic comparison of core genes in paired carriage isolates

**Fig. 1:**
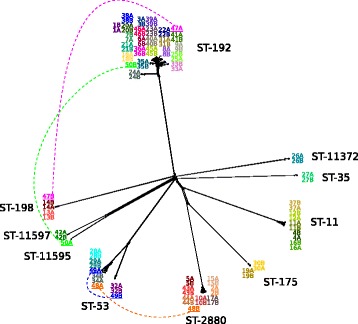
Phylogeny of paired meningococcal carriage isolates. Splits tree based on allelic differences in *N. meningitidis* core genome MLST genes. *Color codes* show paired isolates from the same individual. A and B indicate time points, approximately 2 months apart. Isolates from individuals where different strains were found at the two time points are underlined and connected with dashed lines. ST = sequence type

A phylogenetic comparison of all carriage isolates, based on cgMLST allelic distance matrices, is presented as a Neighbor-net network in Fig. 1. The majority of the paired isolates from the same individual were closely related to each other, with the exception of the isolates from the four individuals where a change of strain was evident (underlined in Fig. 1). The average and median number of allelic differences between paired isolates of the same strain was 35, with a range from 11 to 84 (Additional file 1: Table S1). In the individuals with a change of ST (nos. 47, 48 and 50), allelic differences between the isolates were discovered in 1509, 1461 and 1508 of the 1605 genes of the *N. meningitidis* cgMLST, respectively. In the fourth individual with a change of strain (no. 49), but where the ST remained the same, the number of allelic differences between the isolates was 475 (Additional file 1: Table S1).

Results from further analysis with conventional PCR, targeting the seven housekeeping genes of MLST, as well as the *porA* and *fetA* genes, of the available intermediate isolates from individuals carrying different strains during the two months study period are shown in Table 2. This revealed that individual no. 47 carried three different STs during the period. It also shows that there was two different *porA*- and *fetA*-profiles in the isolates from individual no. 49, giving further support to that these represent two different strains, although the ST remained the same.

**Table 2:** Sequence type (ST), *porA* variant and *fetA* variant of meningococcal carriage isolates from individuals carrying different strains

ID	Time point in weeks^a^	ST	PorA	FetA
47	0 (47A)	192	18-11,42-1	-
	1	192	18-11,42-1	-
	2	192	18-11,42-1	-
	3	192	18-11,42-1	-
	4	192	18-11,42-1	-
	6	2880	5-1,10-8	F1-3
	9 (47B)	198	18.25-11	F5-5
48	0 (48A)	53	7,30-2	F2-28
	3	53	7,30-2	F2-28
	9 (48B)	2880	5-1,10-8	F1-3
49	0 (49A)	53	7.3	F1-7
	1	53	7.3	F1-7
	2	53	7.3	F1-7
	4	53	7.3	F1-7
	5	53	7.3	F1-7
	6	53	7.3	F1-7
	7	53	7.3	F1-7
	8	53	7.3	F1-7
	9 (49B)	53	7-11.30-1	F3-69
50	0 (50A)	11595	18-1,3	F4-72
	2	11595	18-1,3	F4-72
	3	11595	18-1,3	F4-72
	4	11595	18-1,3	F4-72
	6	11595	18-1,3	F4-72
	7	11595	18-1,3	F4-72
	8	11595	18-1,3	F4-72
	9 (50B)	192	18-1,42-1	-

^a^In parentheses, the number identifies the individual and the letters A and B identifies the paired isolates used in the whole genome analysis

Within each ST, there was limited variation and, with the exception of some isolates in ST-53 and ST-192, all isolates within each ST were clustered together (Fig. 1). On average, the number of the 1605 cgMLST genes with allelic differences between the paired isolates from the same STs was 35 for ST-11, 127 for ST-53, 36 for ST-192 and 32 for ST-2880 (Additional file 3: Table S2A-D). When comparing the isolates from different individuals within the same ST, the average increased to 62, 296, 74 and 41 for ST-11, ST-53, ST-192 and ST-2880, respectively (Additional file 3: Table S2A-D). In ST-53, the isolates were divided into two different subclusters (Fig. 1 and Table 3), with individual no. 49 carrying meningococci belonging to each of the two subclusters at different time points. The average number of allelic differences in the 1605 cgMLST genes between the different isolates within each of the two ST-53 subcluster was 34 and 117, whereas the average number of allelic difference between the isolates across the two subclusters was 472 (Additional file 3: Table S2B).

**Table 3:**
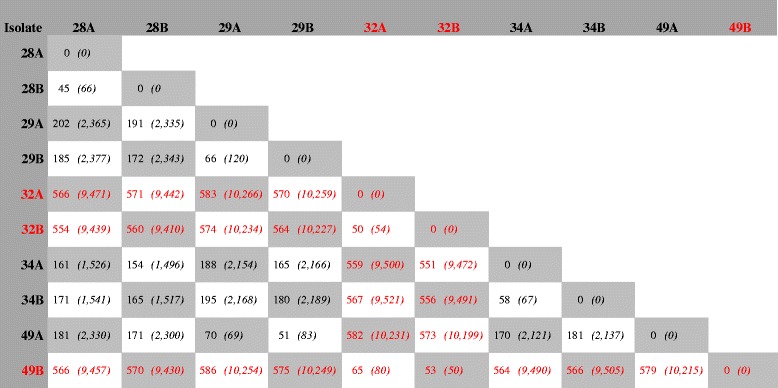
Whole genome comparison of meningococcal carriage isolates in sequence type 53

^a^Allelic differences based on all 2701 annotated genes accessible in Genome Comparator on pubMLST.org as of 27 Oct 2016 (*n* = 2701). Number of nucleotide differences based on the common genome of the 10 isolates, excluding genes that were not present in all isolates

Number of allelic and nucleotide (in italic) differences across the whole genome^a^ between isolates taken from the same individuals at time points A and B. The different colors (black and red) indicate the two subslusters of ST-53

In ST-192, two paired isolates (24A and 24B) were found breaking off the main branch further away from the central cluster where all the other isolates diverged (Fig. 1). Within the main cluster, the paired isolates were generally found most closely related, apart from the isolates belonging to individual no. 9, which shared more common alleles with closely located isolates obtained from other individuals. Isolate 9A shared more allelic variants with multiple isolates, including 25A, 25B, 41A and 41B, than with isolate 9B, which shared more allelic variant with isolates like 38B, 39B and 40A (Additional file 3: Table S2C).

BAPS analyses identified 3, 2, 3, and 2 subclusters, respectively, among the ST-11, ST-53, ST-192 and ST-2880 isolates (Additional file 2: Figure S1 A-D). Isolates from the same individual usually belonged to the same subcluster, except for those from individuals no. 49 (ST-53) and no. 9 (ST-192), confirming the cgMLST results. In ST-2880, however, BAPS distinguished the first isolate from individual no. 15 as a unique subcluster.

### SNP-based comparison of paired carriage isolates

**Fig. 2:**
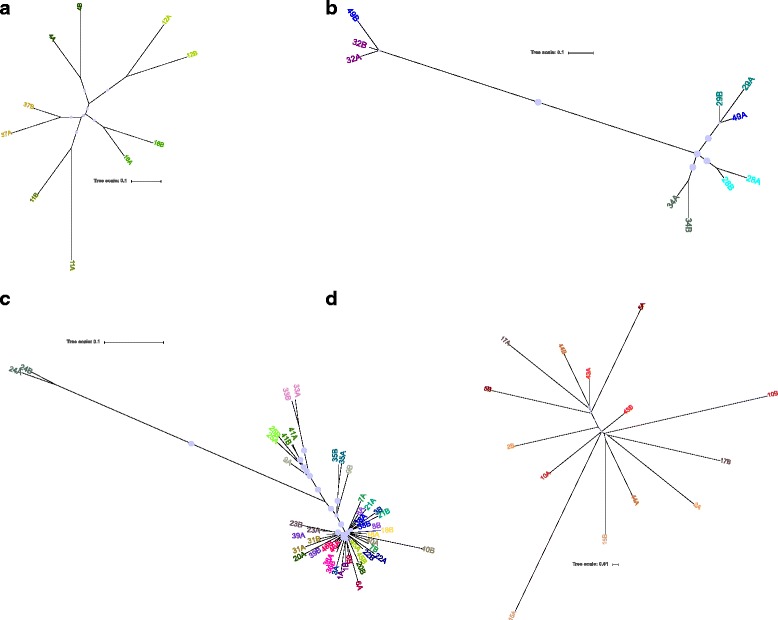
Phylogeny of paired meningococcal carriage isolates per sequence type. Splits tree based on single nucleotide polymorphism (SNP) in *N. meningitidis* core genome MLST genes (*N. meningitidis* cgMLST v1.0, available at pubMLST.org). Panel (**a**), (**b**), (**c**) and (**d**) show the most frequent sequence types (ST) ST-11, ST-53, ST-192 and ST-2880, respectively. *Color codes* show paired isolates from the same individual. A and B indicate time point, approximately two months apart

To compare cgMLST with SNP-based phylogenetic inference, we additionally computed trees based on SNPs in the cgMLST genes after removal of areas with presumed recombination. Trees for each of the STs with 4 or more pairs of isolates are shown in Fig. 2a-d. Overall, the same pattern was seen as in comparison based on allelic differences. The distance range within each ST was similar in ST-11, ST-192 and ST-2880, whereas the distance between the isolates in ST-53 was considerably greater (Fig. 2a-d), i.e., the distance between the two subclusters of ST-53 became even more apparent using SNP-based analysis (Fig. 2b).

For ST-53, the nucleotide differences between all isolates were calculated for the genome shared by all isolates, without removing areas of recombination (Table 3). This revealed an average of 9798 nucleotide differences between the isolates of the two subclusters, compared to an average of 1402 nucleotide differences between isolates of the same subcluster. Table 3 also shows the disproportion in nucleotide and allelic difference between pairs, highlighting the impact of recombination in the meningococcal genomic variation.

### Most variable genes in paired carriage isolates

All annotated meningococcal genes accessible on pubMLST.org as of 27 Oct 2016 were compared pairwise to find the genes which were most frequently differing between pairs. Pairs where a change of strain had occurred were excluded and the 18 genes that differed in ≥ 30% of paired isolates are shown in Table 4. Among the 18 most frequently changed genes, 8 were found within the core genome, highlighted in bold in Table 4. The gene with most frequent genetic change was *pilE*, differing in 85% of the paired isolates. Three of the genes were encoding hypothetical proteins and 2 were putative enzymes, but the majority of genes were expressing surface exposed molecules, like pilin and other membrane proteins.

**Table 4:** Overview of whole genome^a^ genes with most frequent within-host genetic changes in paired meningococcal carriage isolates. Genes belonging to the core genome^b^ are highlighted in bold italic

NEIS no	Gene	Gene product	Function	% of pairs^c^ with genetic difference	Mechanisms and percent of pairs^d^ with genetic difference				
				(*n* = 46)	All STs (*n* = 46)	ST-11 (*n* = 5)	ST-53 (*n* = 4)	ST-192 (*n* = 23)	ST-2880 (*n* = 7)
NEIS0210	*pilE*	pilin	Pilin	85%	GC 85%	no change	GC 75%	GC 96%	GC 100%
pilS	*pilS*	pilin	Pilin	76%	GC 76%	no change	GC 75%	GC 96%	GC 71%
NEIS2649	-	hypothetical protein	Unknown	74%	PM 37%, R 37%	PM 80%, R 20%	R 25%	PM 39%, R 39%	R 57%, PM 14%
NEIS1310	*modA*	type III restriction/modification system methyltransferase	DNA repair	70%	PV 70%	PV 100%	no change	PV 83%	PV 71%
NEIS1403	*opaB*	opacity protein B	Outer membrane component	63%	PV 59%, PM 2%, R 2%	PV 20%	PV 50%	PV 78%	PV 29%
NEIS1719	*opa1800*	outer membrane protein	Outer membrane component	61%	PV 57%, R 9%	PV 20%	PV 50%	PV 78%, R 9%	PV 29%
***NEIS1655***	***relA***	***GTP pyrophosphokinase***	***Housekeeping***	***47%***	***PM 43%, R 4%***	***PM 40%***	***PM 25%***	***PM 43%, R 4%***	***PM 57%***
NEIS0400	*pglH*	glycosyltransferase	Protein glycosylation system	46%	PV 46%	PV 60%	no change	PV 74%	PV 14%
NEIS0401	*pglG*	glycosyltransferase	Protein glycosylation system	44%	PV 37%, R 7%	PV 40%, R 20%	PV 50%	PV 57%, R 9%	no change
***NEIS0380***	***pglI***	***O-acetyltransferase***	***Protein glycosylation system***	***41%***	***PV 41%***	***PV 60%***	***gene absent***	***PV 52%***	***PV 43%***
***NEIS1829***	***tspA***	***neisseria-specific antigen protein***	***Outer membrane component***	***41%***	***R 33%, D 15%, PM 2%, I 2%***	***no change***	***D 25%, R 25%***	***R 61%, D 22%, PM 4%***	***no change***
***NEIS1750***	***-***	***hypothetical protein***	***Unknown***	***37%***	***PV 35%, PM 2%***	***PV 20%***	***no change***	***PV 43%, PM 4%***	***PV 29%***
NEIS1946	*hpuA*	haemoglobin-haptoglobin utilisation protein	Outer membrane component	35%	PV 33%, PM 2%, R 2%	PV 40%	no change	PV 52%, PM 4%, R 4%	no change
***NEIS1288***	***-***	***putative aldehyde dehydrogenase***	***Putative housekeeping***	***35%***	***R 22%, PM 13%***	***PM 20%, R 20%***	***PM 75%***	***R 26%***	***PM 29%, R 29%***
***NEIS1902***	***lgtA***	***lacto-N-neotetraose biosynthesis glycosyl transferase***	***LOS biosynthesis***	***34%***	***PV 30%, D 4%***	***no change***	***no change***	***PV 61%, D 9%***	***no change***
NEIS1418	-	putative membrane peptidase	Putative housekeeping	33%	D 13%, R 11%, PM 9%	PM 20%	PM 25%, D 25%	D 22%, PM 9%, R 4%	R 57%
***NEIS1852***	***emrB***	***multidrug resistance translocase***	***Outer membrane component***	***33%***	***PM 24%, R 9%***	***PM 60%***	***PM 50%, R 25%***	***PM 9%***	***PM 29%, R 29%***

*D* Deletion, *GC* gene conversion, *I* insertion, *PM* point mutation, *PV* phase variation, *R* recombination

^a^All 2701 annotated genes accessible in Genome Comparator on pubMLST.org as of 27 Oct 2016

^b^1605 genes defined as the core genome (*N. meningitidis* cgMLST v1.0) in the database pubMLST.org

^c^Pairs with any genetic differences. Pairs from individuals carrying different strains at the two time points were excluded from the analysis

^d^In some pairs, more than one mechanism of genetic change were observed

The different STs showed variation with regard to which genes that were most frequently changed (Additional file 4: Table S3A-D). In ST-192, *lgtA* changed by phase variation in 61% of the paired isolates, but were not changed in any of the pairs in ST-11, ST-53 and ST-2880, as these isolates do not have any phase variable tracts in *lgtA. pilE* and *pilS* changed in at least 75 and 71%, respectively, of the paired isolates belonging to ST-53, ST-192 and ST-2880, whereas no changes were seen in any of the pairs in ST-11. *pglG* changed in at least 40% of the paired isolates except for in ST-2880, where the gene was absent.

### Core genome genes most frequently changed in paired isolates

**Fig. 3:**
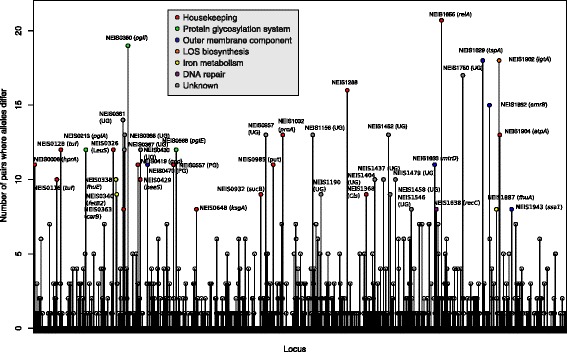
Frequency of changes in *N. meningitidis* core genome MLST genes between paired meningococcal carriage isolates. *Vertical lines* show frequency of pairs with allelic differences. Gene name and color coding according to functional group are added to the genes that most frequently differed (in ≥8 pairs). UD = undesignated gene, PG = putative gene function

A total of 566 cgMLST genes was changed in one or more of the 46 pairs. The most frequently changed cgMLST genes between paired isolates (changed in ≥8 pairs) were spread across the genome. These are marked by color according to functional group in Fig. 3. The most frequently changed cgMLST gene, NEIS1655 (*relA)* was changed in 21 of 46 pairs.

### Mechanisms of genetic variation in frequently changed genes

Alleles can differ by a single or multiple nucleotides. Single nucleotide changes are presumably caused by point mutation, whereas multiple nucleotide differences and insertions/deletions are most likely due to recombination events with a different strain or species. Phase variation, as determined by the presence of repeating nucleotides or short repeated sequences of variable lengths, was the most common mechanism behind the genetic difference seen between isolates, followed by recombination and mutation.

Phase variation was found in genes belonging to the *O*-linked protein glycosylation system (*pglH, pglG* and *pglI*), lipooligosaccharide biosynthesis (*lgtA*) and outer membrane components (*opaB, opa1800* and *hpuA*). Pilus antigenic variation is due to changes in the *pilE* gene that results from introduction of segments of non-expressed *pilS* cassettes into the locus by recombination events, known as gene conversion [53, 54]. This was seen in the majority of pairs, except for ST-11. It has recently been shown that a limited number of clonal complexes, including cc11, express the conserved class II *pilE* gene that do not undergo antigenic variation [55]. Recombination was seen in two putative housekeeping genes at a frequency of 20%, and occurred at a frequency of 2–6% in multiple other genes (Table 4). In several genes, the genetic differences were seen only as single nucleotide changes or recombination in the same position(s) in more than one pair (Additional file 4: Table S3A-D). For example, in the hypothetical protein NEIS2649, GTP pyrophosphokinase NEIS1655, and multidrug resistance translocase NEIS1852, the nucleotide differed either between G and T or A and C, but there was no pattern across the samples suggestive of a sample mix-up and the reads were consistent with the same nucleotide. The nucleotide change resulted in a change of amino acid sequence in all three genes.

Gene changes assigned to the recombination mechanism involved multiple nucleotide differences within regions of the genes varying from 4 to 762 nucleotides. All nucleotide differences assigned as point mutations appeared as a single mutation within each gene, except for in NEIS1655 where two SNPs were located 1911 nucleotides apart. The positions of single nucleotide changes were often the same across different STs, suggesting that they might be however due to recombination of sequences differing only by a single nucleotide rather than point mutations.

## Discussion

We present here the WGS data of within-host paired longitudinal meningococcal carriage isolates, taken on average two months apart, from healthy individuals in Ethiopia. The genetic changes observed in this study are believed to be random or individual dependent, as no known driving forces were present, except for the two individuals carrying serogroup W strains and receiving a conjugate vaccine containing serogroup W. No obvious difference in genetic changes between the vaccinated and non-vaccinated serogroup W carriers was observed, and none of these individuals carried different strains. However, the numbers are too low to draw conclusion.

The majority of isolates were non-groupable, most of them containing the *cnl* locus, lacking the possibility to express capsule, whereas some non-groupable isolates lacked some, but not all genes necessary for capsule synthesis (Table 1). Although the capsule has been seen as an important factor for transmission success, as the meningococcus is sensitive to drying out [56], the high prevalence of meningococcal carrier strains harboring the *cnl* locus provides evidence that the bacterial capsule is not required for person-to-person transmission. The finding of different isolates in the same individual over time, with likely strain replacement of one isolate harboring the *cnl* locus, belonging to ST-11595, with another isolate harboring the *cnl* locus, belonging to ST-192, also supports this conclusion.

WGS gives a much better resolution of genomic relationships and here we revealed a difference in strains in an individual that would not have been discovered by traditional MLST typing. The difference in 475 cgMLST genes between the two isolates from individual no. 49 is more than ten times higher than the number of differences typically seen in pairs of the same strain, suggesting that the differences are highly unlikely caused by genetic changes within the host. The finding of equally many allelic differences between unrelated isolates from different STs (523 and 522 cgMLST genes in isolate 50A from ST-11595 compared to isolates 42A and 42B from ST-11597, respectively) also indicate that the difference seen between the isolates from individual no. 49 is caused by colonization with a different strain, not with-in host genetic changes in one third of the cgMLST genes. In addition, within ST-53 the isolates belonged to two different subclusters based on cgMLST. Different *porA*- and *fetA*-profiles, which are widely used in meningococcal classification in addition to MLST, also indicated that there was a difference between these two ST-53 isolates. This illustrates the limitation of traditional MLST, when classification and comparison are based only on a few selected genes. WGS and cgMLST provide much finer tools for identification of meningococcal sublineages and has already been very valuable in resolving global epidemiology, and the unravelling of the spread of serogroup W, ST-11, showed that how multiple sublineages of the same clonal complex may coexist within the same area [57, 58].

Analyzing paired carriage isolates gives insight into the genetic evolution of the bacterium when residing in its natural habitat. The isolates were taken only 6-9 weeks apart, but genetic and phenotypic changes in the meningococcus have been shown to happen over a very short time in vivo, directly impacting the adaptation of the bacterium to evade the host immune system and increases its virulence [59]. The number of genes with allelic differences between isolates from different individuals within the same ST was about twice as many as the differences between paired isolates from the same carrier. Estimated mean duration of meningococcal carriage ranges from 3.4 to 11.7 months [24, 25, 60] and the increased difference from within-host to between-host changes is likely explained by increased time from acquisition to sampling, compared to the two months between the two samplings in the study. A newly published study from the Netherlands revealed progressively acquired mutations and/or recombination events in three carriers harboring the same strain at three time-points in an 8-month period, again underlining the evolution and adaptation of the bacteria during carriage [61]. As in our analysis, incomplete genes were excluded, we may have underestimated the number of differences.

A recent study looking at within-host evolution by genomic comparison of throat and blood strain pairs from four patients with meningococcal disease, also found changes in *pilE, modA* and *pglI* [62], which were among the most frequently changed genes in our study. Additional genes identified in the study by Klughammer et al. [62], like *pilC1* and *fetA*, were also seen to change in our study, but to a lesser extent, in 15 and 11% of paired isolates, respectively. The authors hypothesized that genomic variants arise during carriage and that invasive variants occasionally emerge and cause disease [62]. In contrast, no further genetic changes seem to be necessary for the bacteria to cross the blood-brain barrier [63].

Phase variation is a mechanism with high frequency, resulting in reversible regulation of expression [33] and therefore expected to occur within short-term carriage. A recent study identified 277 phase variable gene candidates in the meningococcus and classified them as strong, moderate or weak based on repeat variability and intra-strain phylogenetic relationship in 20 available *N. meningitidis* genomes [64]. We and others have identified phase variation in genes involved in surface exposed structures and membrane components such as, opacity proteins [65], lipopolysaccharide biosynthesis [66], and pilin/protein glycosylation [67, 68], but also restriction/modification systems [69] and a hemoglobin receptor [70]. Phase variation in pilin glycosylation is seen in patterns of homopolymeric tracts with ≥7 repeating nucleotides, as is phase variation in the lipopolysaccharide glycosyltransferases [66]. In our study we also found phase variation as tetrameric repeat units in the restriction-modification system responsible for defense against invasion of foreign DNA [69] and pentameric repeat units in the outer membrane proteins [71]. Phase variation is known to cause rapid changes in the bacteria, both in the laboratory and within the host, and differences due to phase variation were seen in up to 80% of pairs within the 9 weeks of the study. In fact, comparative genomic studies after an accidental human passage and disease revealed increased changes in phase variable genes, and the authors suggested that this emphasized the importance of phase variation for in vivo adaption of meningococci [59].

The much higher rate of recombination to mutation in meningococci makes phylogenetic analyses difficult, as SNP-based analysis will overestimate the number of mutations and the evolutionary distance between isolates if recombination is not taken into consideration [72, 73]. Analysis using allelic differences on a gene-by-gene basis, on the other hand, treats all genetic changes within a gene as one event and are more suited in highly recombining bacteria [74]. However, an allelic approach runs the risk of underestimating the number of mutations and the genetic distances. Therefore, allelic comparisons were used for analysis amongst different STs, where the differences in SNP are expected to be high (Fig. 1), whereas SNP-based analyses with higher resolution were used for phylogenetic analysis within STs, after excluding presumed recombination events (Fig. 2a-d).

The findings of single nucleotide changes or recombination at specific positions in some genes do not appear to be random or due to technical errors. The pattern and position of the nucleotide changes were consistent across different STs. Most allelic variants were seen in more than one pair and in already annotated alleles available in BIGSdb, suggesting they may be due to recombination of sequences differing only in a single nucleotide position (marked with an asterisk in Additional file 4:. Table S3A-D) rather than point mutations. In the same genes, recombination was found in the same area in other isolates, indicating that these areas are hot spots for recombination.

As we only picked single colonies from the plates where the swabs were cultivated, this study does not allow to determine whether strain heterogeneity was present on any sample time. Thus, it is not possible to conclude whether the difference in strains observed were actual replacement or due to concomitant carriage where one ST was sampled at one time and the other ST was sampled the next time. A study picking up to 20 individual colonies from the original plate, found carriage of multiple clones in about 1.4% of meningococcal carriers [9]. This study used 15 variable-number tandem repeats (VNTR) loci for differentiating between clones, a method more likely to underestimate than overestimate the number of clones, as compared to WGS. In the individual where three different strains were identified in five weeks, concomitant carriage of all three strains cannot be excluded and concomitant carriage of at least two of these strains at a given time is highly likely. The high frequency of recombination in some genes also suggests that concomitant carriage of more than one clone is more common than previously believed. This should be further investigated by the use of WGS to analyze multiple colonies from the same sample.

Although we cannot conclude how common strain replacement is in the general population of meningococcal carriers based on this study, as the individuals were chosen based on the availability of two paired meningococcal isolates, the change of strain in 4 out of 50 (8%) of the individuals shows that meningococcal carriage in an African population is dynamic and the likelihood of concomitant carriage is quite high. In comparison, a European study of teenagers in 2008 found that 36.7% of those who remained carriers over a period of 23 weeks acquired a new strain during that period [75]. The same study also found one individual out of 72 who carried 3 different strains over a period of 6 months [75]. However, this study used serological, capsule PCR and pulse field gel electrophoresis (PFGE) analyses, which are less discriminatory than WGS and strain changes within serogroups or PFGE-groups would not have been discovered. Taking the relatively short duration and a lower number of individuals in our study, our findings might be an indication that strain replacement is more common in this population. A recent study in several African countries, also using traditional MLST typing, found that 4% carried different strains on visits about one month apart [25]. Further studies using WGS are therefore necessary to understand the dynamics and carriage of heterogeneous strains, and to what extent there are differences within different host populations or strains of *Neisseria.*

## Conclusions

High resolution genome-wide sequence typing is necessary to resolve the diversity of meningococcal isolates and reveals genetic differences not discovered by traditional typing schemes. WGS should be the method of choice for strain characterization, as the technology has improved and costs decreased. The most frequently changed genes were genes belonging to the pilin family, the restriction/modification system, opacity proteins and genes involved in glycosylation. The most frequent mechanisms of change were phase variation and recombination. There were about twice as many allelic differences between unpaired isolates from different individuals as in paired isolates within each ST, which may be explained by difference in time or by distinct, unknown selection pressure driven by the immune systems of these individuals.

## Additional files

Additional file 1: Table S1. Number and percent of allelic differences in cgMLST genes^a^ and isolate ID in BIGSdb. (DOCX 26 kb)

Additional file 2: Figure S1. Bayesian analysis of population structure (BAPS) clusters of paired meningococcal carriage isolates per sequence type. Neighbor-net splits trees of allelic differences in *N. meningitidis* core genome MLST genes (*N. meningitidis* cgMLST v1.0, available at pubMLST.org). Panel A, B, C and D show sequence types (ST) ST-11, ST-53, ST-192 and ST-2880, respectively. BAPS clusters are highlighted by circles and numbered sequentially. Paired isolates from the same individual that have been assigned to different BAPS clusters are underlined and connected with dashed lines. (ZIP 56 kb)

Additional file 3: Table S2A-D. Comparison of meningococcal carriage isolates in sequence types 11, 53, 192 and 2880. Number of allelic differences in the 1,605 genes of the *N. meningitidis* core genome^*^. (DOCX 55 kb)

Additional file 4: Table S3A-D. Mechanism of genetic change in paired meningococcal carriage isolates in sequence types 11, 53, 192 and 2880. (DOCX 59 kb)

## Abbreviations

BIGSdb: Bacterial isolate genome sequence database
Cg: Core genome
cgMLST: Core genome multilocus sequence typing
MLST: Multilocus sequence typing
NG: Non-groupable
NIPH: Norwegian institute of public health
PFGE: Pulse field gel electrophoresis
SNP: Single nucleotide polymorphism
ST: Sequence type
VNTR: Variable-number tandem repeats
WGS: Whole genome sequences

## References

[1] Broome CV (1986). The carrier state: *Neisseria meningitidis*. J Antimicrob Chemother.

[2] Trotter CL, Greenwood BM (2007). Meningococcal carriage in the African meningitis belt. Lancet Infect Dis.

[3] Yazdankhah SP, Caugant DA (2004). *Neisseria meningitidis*: an overview of the carriage state. J Med Microbiol.

[4] Edwards EA, Devine LF, Sengbusch GH, Ward HW (1977). Immunological investigations of meningococcal disease. III. Brevity of group C acquisition prior to disease occurrence. Scand J Infect Dis.

[5] Tzeng YL, Stephens DS (2000). Epidemiology and pathogenesis of *Neisseria meningitidis*. Microbes Infect.

[6] Pollard AJ, Maiden MCJ (eds.). Meningococcal disease. Totowa: Humana Press; 2001.

[7] Brandtzaeg P, van Deuren M (2012). Classification and pathogenesis of meningococcal infections. Methods Mol Biol.

[8] Harrison OB, Claus H, Jiang Y, Bennett JS, Bratcher HB, Jolley KA (2013). Description and nomenclature of *Neisseria meningitidis* capsule locus. Emerg Infect Dis.

[9] Caugant DA, Tzanakaki G, Kriz P (2007). Lessons from meningococcal carriage studies. FEMS Microbiol Rev.

[10] Xu Z, Zhu B, Xu L, Gao Y, Shao Z (2015). First case of *Neisseria meningitidis* capsule null locus infection in China. Infect Dis.

[11] Vogel U, Claus H, von Muller L, Bunjes D, Elias J, Frosch M (2004). Bacteremia in an immunocompromised patient caused by a commensal *Neisseria meningitidis* strain harboring the capsule null locus (*cnl*). J Clin Microbiol.

[12] Hoang LM, Thomas E, Tyler S, Pollard AJ, Stephens G, Gustafson L (2005). Rapid and fatal meningococcal disease due to a strain of *Neisseria meningitidis* containing the capsule null locus. Clin Infect Dis.

[13] Rosenstein NE, Perkins BA, Stephens DS, Popovic T, Hughes JM (2001). Meningococcal disease. N Engl J Med.

[14] Hammerschmidt S, Muller A, Sillmann H, Muhlenhoff M, Borrow R, Fox A (1996). Capsule phase variation in *Neisseria meningitidis* serogroup B by slipped-strand mispairing in the polysialyltransferase gene (*siaD*): correlation with bacterial invasion and the outbreak of meningococcal disease. Mol Microbiol.

[15] Tzeng YL, Thomas J, Stephens DS (2016). Regulation of capsule in *Neisseria meningitidis*. Crit Rev Microbiol.

[16] Mueller JE, Gessner BD (2010). A hypothetical explanatory model for meningococcal meningitis in the African meningitis belt. Int J Infect Dis.

[17] Koutangni T, Boubacar Mainassara H, Mueller JE (2015). Incidence, carriage and case-carrier ratios for meningococcal meningitis in the African meningitis belt: a systematic review and meta-analysis. PLoS One.

[18] Baker M, McNicholas A, Garrett N, Jones N, Stewart J, Koberstein V (2000). Household crowding a major risk factor for epidemic meningococcal disease in Auckland children. Pediatr Infect Dis J.

[19] Pether JVS, Lightfoot NF, Scott RJD, Morgan J, Steele-Perkins AP, Sheard SC (1988). Carriage of *Neisseria meningitidis*: Investigations in a military establishment. Epidemiol Infect.

[20] Coen PG, Tully J, Stuart JM, Ashby D, Viner RM, Booy R (2006). Is it exposure to cigarette smoke or to smokers which increases the risk of meningococcal disease in teenagers?. Int J Epidemiol.

[21] Mueller JE, Yaro S, Madec Y, Somda PK, Idohou RS, Lafourcade BM (2008). Association of respiratory tract infection symptoms and air humidity with meningococcal carriage in Burkina Faso. Trop Med Int Health.

[22] Goldblatt D (2000). Conjugate vaccines. Clin Exp Immunol.

[23] Ala’Aldeen DA, Neal KR, Ait-Tahar K, Nguyen-Van-Tam JS, English A, Falla TJ (2000). Dynamics of meningococcal long-term carriage among university students and their implications for mass vaccination. J Clin Microbiol.

[24] De Wals P, Gilquin C, De Maeyer S, Bouckaert A, Noel A, Lechat MF (1983). Longitudinal study of asymptomatic meningococcal carriage in two Belgian populations of schoolchildren. J Infect.

[25] MenAfriCar consortium (2016). Household transmission of *Neisseria meningitidis* in the African meningitis belt: a longitudinal cohort study. Lancet Glob Health.

[26] Kwong JC, McCallum N, Sintchenko V, Howden BP (2015). Whole genome sequencing in clinical and public health microbiology. Pathology.

[27] Maiden MC, van Rensburg MJ J, Bray JE, Earle SG, Ford SA, Jolley KA (2013). MLST revisited: the gene-by-gene approach to bacterial genomics. Nat Rev Microbiol.

[28] Rotman E, Seifert HS (2014). The genetics of *Neisseria* species. Annu Rev Genet.

[29] Feavers IM (2000). ABC of meningococcal diversity. Nature.

[30] Davidsen T, Rodland EA, Lagesen K, Seeberg E, Rognes T, Tonjum T (2004). Biased distribution of DNA uptake sequences towards genome maintenance genes. Nucleic Acids Res.

[31] Feil EJ, Maiden MC, Achtman M, Spratt BG (1999). The relative contributions of recombination and mutation to the divergence of clones of *Neisseria meningitidis*. Mol Biol Evol.

[32] Jolley KA, Wilson DJ, Kriz P, McVean G, Maiden MC (2005). The influence of mutation, recombination, population history, and selection on patterns of genetic diversity in *Neisseria meningitidis*. Mol Biol Evol.

[33] Henderson IR, Owen P, Nataro JP (1999). Molecular switches--the ON and OFF of bacterial phase variation. Mol Microbiol.

[34] Barnes GK, Kristiansen PA, Beyene D, Workalemahu B, Fissiha P, Merdekios B (2016). Prevalence and epidemiology of meningococcal carriage in Southern Ethiopia prior to implementation of MenAfriVac, a conjugate vaccine. BMC Infect Dis.

[35] Riou JY, Guibourdenche M (1993). Methodes de laboratoire *Neisseria* et *Branhamella*.

[36] Craven DE, Frasch CE, Robbins JB, Feldman HA (1978). Serogroup identification of *Neisseria meningitidis*: comparison of an antiserum agar method with bacterial slide agglutination. J Clin Microbiol.

[37] Maiden MC, Bygraves JA, Feil E, Morelli G, Russell JE, Urwin R (1998). Multilocus sequence typing: a portable approach to the identification of clones within populations of pathogenic microorganisms. Proc Natl Acad Sci U S A.

[38] Bolger AM, Lohse M, Usadel B (2014). Trimmomatic: a flexible trimmer for Illumina sequence data. Bioinformatics.

[39] Bankevich A, Nurk S, Antipov D, Gurevich AA, Dvorkin M, Kulikov AS (2012). SPAdes: a new genome assembly algorithm and its applications to single-cell sequencing. J Comput Biol.

[40] Davis MP, van Dongen S, Abreu-Goodger C, Bartonicek N, Enright AJ (2013). Kraken: a set of tools for quality control and analysis of high-throughput sequence data. Methods.

[41] Jolley KA, Maiden MC (2010). BIGSdb: Scalable analysis of bacterial genome variation at the population level. BMC Bioinf.

[42] Bratcher HB, Corton C, Jolley KA, Parkhill J, Maiden MC (2014). A gene-by-gene population genomics platform: de novo assembly, annotation and genealogical analysis of 108 representative *Neisseria meningitidis* genomes. BMC Genomics.

[43] Bryant D, Moulton V (2004). Neighbor-net: an agglomerative method for the construction of phylogenetic networks. Mol Biol Evol.

[44] Huson DH, Bryant D (2006). Application of phylogenetic networks in evolutionary studies. Mol Biol Evol.

[45] Cheng L, Connor TR, Sirén J, Aanensen DM, Corander J (2013). Hierarchical and spatially explicit clustering of DNA sequences with BAPS software. Mol Biol Evol.

[46] Treangen TJ, Ondov BD, Koren S, Phillippy AM (2014). The Harvest suite for rapid core-genome alignment and visualization of thousands of intraspecific microbial genomes. Genome Biol.

[47] Croucher NJ, Page AJ, Connor TR, Delaney AJ, Keane JA, Bentley SD (2015). Rapid phylogenetic analysis of large samples of recombinant bacterial whole genome sequences using Gubbins. Nucleic Acids Res.

[48] Stamatakis A (2014). RAxML version 8: a tool for phylogenetic analysis and post-analysis of large phylogenies. Bioinformatics.

[49] Letunic I, Bork P (2016). Interactive tree of life (iTOL) v3: an online tool for the display and annotation of phylogenetic and other trees. Nucleic Acids Res.

[50] Kumar S, Stecher G, Tamura K (2016). MEGA7: molecular evolutionary genetics analysis version 7.0 for bigger datasets. Mol Biol Evol.

[51] Quail MA, Smith M, Coupland P, Otto TD, Harris SR, Connor TR (2012). A tale of three next generation sequencing platforms: comparison of Ion torrent, pacific biosciences and illumina MiSeq sequencers. BMC Genomics.

[52] Boratyn GM, Camacho C, Cooper PS, Coulouris G, Fong A, Ma N (2013). BLAST: a more efficient report with usability improvements. Nucleic Acids Res.

[53] Hagblom P, Segal E, Billyard E, So M (1985). Intragenic recombination leads to pilus antigenic variation in *Neisseria gonorrhoeae*. Nature.

[54] Segal E, Billyard E, So M, Storzbach S, Meyer TF (1985). Role of chromosomal rearrangement in *N. gonorrhoeae* pilus phase variation. Cell.

[55] Wormann ME, Horien CL, Bennett JS, Jolley KA, Maiden MC, Tang CM (2014). Sequence, distribution and chromosomal context of class I and class II pilin genes of *Neisseria meningitidis* identified in whole genome sequences. BMC Genomics.

[56] Weber MV, Claus H, Maiden MC, Frosch M, Vogel U (2006). Genetic mechanisms for loss of encapsulation in polysialyltransferase-gene-positive meningococci isolated from healthy carriers. Int J Med Microbiol.

[57] Lucidarme J, Hill DM, Bratcher HB, Gray SJ, du Plessis M, Tsang RS (2015). Genomic resolution of an aggressive, widespread, diverse and expanding meningococcal serogroup B, C and W lineage. J Infect.

[58] Mustapha MM, Marsh JW, Krauland MG, Fernandez JO, de Lemos APS, Dunning Hotopp JC (2015). Genomic epidemiology of hypervirulent serogroup W, ST-11 *Neisseria meningitidis*. EBioMedicine.

[59] Omer H, Rose G, Jolley KA, Frapy E, Zahar JR, Maiden MC (2011). Genotypic and phenotypic modifications of *Neisseria meningitidis* after an accidental human passage. PLoS One.

[60] Gold R, Goldschneider I, Lepow ML, Draper TF, Randolph M (1978). Carriage of *Neisseria meningitidis* and *Neisseria lactamica* in infants and children. J Infect Dis.

[61] van Ravenhorst MB, Bijlsma MW, van Houten MA, Struben VMD, Anderson AS, Eiden J et al. Meningococcal carriage in Dutch adolescents and young adults; a cross-sectional and longitudinal cohort study. Clin Microbiol Infect. 2017. Epub ahead of print. 10.1016/j.cmi.2017.02.008 28192234

[62] Klughammer J, Dittrich M, Blom J, Mitesser V, Vogel U, Frosch M (2017). Comparative genome sequencing reveals within-host genetic changes in *neisseria meningitidis* during invasive disease. PLoS One.

[63] Lees JA, Kremer PHC, Manso AS, Croucher NJ, Ferwerda B, Valls Seron M et al. Large scale genomic analysis shows no evidence for pathogen adaptation between the blood and cerebrospinal fluid niches during bacterial meningitis. Microbial Genomics. 2017. DOI 10.1099/mgen.0.000103 Epub ahead of print. 10.1099/mgen.0.000103 PMC5361624 28348877

[64] Siena E, D’Aurizio R, Riley D, Tettelin H, Guidotti S, Torricelli G, Moxon ER, Medini D (2016). In-silico prediction and deep-DNA sequencing validation indicate phase variation in 115 *Neisseria meningitidis* genes. BMC Genomics.

[65] Stern A, Brown M, Nickel P, Meyer TF (1986). Opacity genes in *Neisseria gonorrhoeae*: control of phase and antigenic variation. Cell.

[66] Jennings MP, Srikhanta YN, Moxon ER, Kramer M, Poolman JT, Kuipers B (1999). The genetic basis of the phase variation repertoire of lipopolysaccharide immunotypes in *Neisseria meningitidis*. Microbiol.

[67] Banerjee A, Ghosh SK (2003). The role of pilin glycan in neisserial pathogenesis. Mol Cell Biochem.

[68] Power PM, Roddam LF, Rutter K, Fitzpatrick SZ, Srikhanta YN, Jennings MP (2003). Genetic characterization of pilin glycosylation and phase variation in *Neisseria meningitidis*. Mol Microbiol.

[69] Fox KL, Srikhanta YN, Jennings MP (2007). Phase variable type III restriction-modification systems of host-adapted bacterial pathogens. Mol Microbiol.

[70] Tauseef I, Harrison OB, Wooldridge KG, Feavers IM, Neal KR, Gray SJ (2011). Influence of the combination and phase variation status of the haemoglobin receptors HmbR and HpuAB on meningococcal virulence. Microbiol.

[71] Kupsch EM, Knepper B, Kuroki T, Heuer I, Meyer TF (1993). Variable opacity (Opa) outer membrane proteins account for the cell tropisms displayed by *Neisseria gonorrhoeae* for human leukocytes and epithelial cells. EMBO J.

[72] Schierup MH, Hein J (2000). Consequences of recombination on traditional phylogenetic analysis. Genetics.

[73] Awadalla P (2003). The evolutionary genomics of pathogen recombination. Nat Rev Genet.

[74] Didelot X, Maiden MC (2010). Impact of recombination on bacterial evolution. Trends Microbiol.

[75] Glitza IC, Ehrhard I, Muller-Pebody B, Reintjes R, Breuer T, Ammon A (2008). Longitudinal study of meningococcal carrier rates in teenagers. Int J Hyg Environ Health.

