# Supplementary material for: Whole genome sequencing reveals within-host genetic changes in paired meningococcal carriage isolates from Ethiopia
Source: BMC Genomics. 2017 May 25;18:407. doi: 10.1186/s12864-017-3806-3 (PMC5445459; doi:10.1186/s12864-017-3806-3)
Supplement: Supplementary file 1 — Number and percent of allelic differences in cgMLST genesa and isolate ID in BIGSdb. (DOCX 26 kb) [file 12864_2017_3806_MOESM1_ESM.docx]

**Additional file 1: Table S1**

**Number and percent of allelic differences in cgMLST genes^a^ and isolate ID in BIGSdb^b^**

| **Individual** | **Sex** | **Age** | **Weeks between** | **cgMLST** | |  | | **BIGSdb isolate ID** | |
| --- | --- | --- | --- | --- | --- | --- | --- | --- | --- |
|  |  |  | **sample A and B** | **No. of diff** | **Percent diff.** | | **Time point A** | | **Time point B** |
| **1** | M | 10 | 8 | 34 | (2%) |  | | 44913 | 44914 |
| **2** | M | 3 | 9 | 40 | (2%) |  | | 41874 | 41876 |
| **3** | M | 7 | 8 | 42 | (3%) |  | | 44915 | 44916 |
| **4** | M | 4 | 7 | 37 | (2%) |  | | 41877 | 41878 |
| **5** | F | 9 | 9 | 28 | (2%) |  | | 41879 | 41880 |
| **6** | M | 15 | 7 | 28 | (2%) |  | | 44917 | 44918 |
| **7** | F | 14 | 9 | 38 | (2%) |  | | 44919 | 44920 |
| **8** | F | 3 | 9 | 26 | (2%) |  | | 44921 | 47212 |
| **9** | F | 12 | 9 | 84 | (5%) |  | | 45082 | 44923 |
| **10** | M | 9 | 7 | 33 | (2%) |  | | 41881 | 41883 |
| **11** | F | 10 | 8 | 47 | (3%) |  | | 41884 | 41885 |
| **12** | M | 8 | 9 | 40 | (2%) |  | | 44926 | 44927 |
| **13** | F | 13 | 9 | 24 | (1%) |  | | 41886 | 41888 |
| **14** | F | 8 | 9 | 21 | (1%) |  | | 41889 | 41891 |
| **15** | M | 6 | 6 | 52 | (3%) |  | | 44930 | 44931 |
| **16** | M | 5 | 9 | 21 | (1%) |  | | 44932 | 44933 |
| **17** | M | 7 | 9 | 31 | (2%) |  | | 44934 | 44935 |
| **18** | M | 18 | 8 | 45 | (3%) |  | | 44936 | 44937 |
| **19** | M | 8 | 7 | 23 | (1%) |  | | 41896 | 41897 |
| **20** | M | 14 | 9 | 36 | (2%) |  | | 44938 | 44939 |
| **21** | M | 11 | 9 | 41 | (3%) |  | | 44940 | 44941 |
| **22** | M | 6 | 8 | 38 | (2%) |  | | 44942 | 44943 |
| **23** | M | 6 | 8 | 36 | (2%) |  | | 44944 | 44945 |
| **24** | F | 28 | 9 | 40 | (2%) |  | | 44946 | 44947 |
| **25** | F | 8 | 9 | 44 | (3%) |  | | 44948 | 44949 |
| **26** | F | 2 | 8 | 51 | (3%) |  | | 44950 | 44951 |
| **27** | F | 28 | 9 | 37 | (2%) |  | | 41901 | 41842 |
| **28** | F | 27 | 6 | 32 | (2%) |  | | 41843 | 41844 |
| **29** | F | 6 | 8 | 55 | (3%) |  | | 42663 | 42665 |
| **30** | F | 4 | 9 | 33 | (2%) |  | | 42666 | 42668 |
| **31** | F | 5 | 8 | 40 | (2%) |  | | 44954 | 44955 |
| **32** | M | 8 | 9 | 27 | (2%) |  | | 42669 | 42671 |
| **33** | F | 27 | 7 | 48 | (3%) |  | | 44956 | 44957 |
| **34** | M | 8 | 8 | 48 | (3%) |  | | 42673 | 42675 |
| **35** | F | 13 | 9 | 36 | (2%) |  | | 44960 | 44899 |
| **36** | F | 20 | 8 | 16 | (1%) |  | | 44900 | 45081 |
| **37** | F | 11 | 8 | 28 | (2%) |  | | 42678 | 42679 |
| **38** | M | 3 | 8 | 20 | (1%) |  | | 45955 | 45956 |
| **39** | M | 13 | 8 | 19 | (1%) |  | | 44901 | 44902 |
| **40** | F | 8 | 9 | 60 | (4%) |  | | 44903 | 44904 |
| **41** | M | 9 | 9 | 15 | (1%) |  | | 44905 | 44906 |
| **42** | M | 12 | 9 | 19 | (1%) |  | | 42680 | 42682 |
| **43** | F | 29 | 8 | 11 | (1%) |  | | 44907 | 44908 |
| **44** | M | 10 | 9 | 28 | (2%) |  | | 42685 | 42686 |
| **45** | M | 6 | 8 | 14 | (1%) |  | | 44909 | 44910 |
| **46** | F | 6 | 8 | 29 | (2%) |  | | 44911 | 44912 |
| **47** | M | 8 | 9 | 1509 | (94%) |  | | 44924 | 44925 |
| **48** | F | 9 | 9 | 1461 | (91%) |  | | 44928 | 44929 |
| **49** | M | 3 | 9 | 475 | (30%) |  | | 44952 | 44953 |
| **50** | F | 29 | 9 | 1508 | (94%) |  | | 44958 | 44959 |

^a^1,605 genes in the *N. meningitidis* cgMLST v1.0 accessible on pubMLST.org as of 27 Oct 2016

^b^Bacterial Isolate Genome Sequence Database, all whole genome sequences are publicly available at pubMLST.org
