# Supplementary figures and images for: Whole genome sequencing reveals within-host genetic changes in paired meningococcal carriage isolates from Ethiopia
Source: BMC Genomics. 2017 May 25;18:407. doi: 10.1186/s12864-017-3806-3 (PMC5445459; doi:10.1186/s12864-017-3806-3)

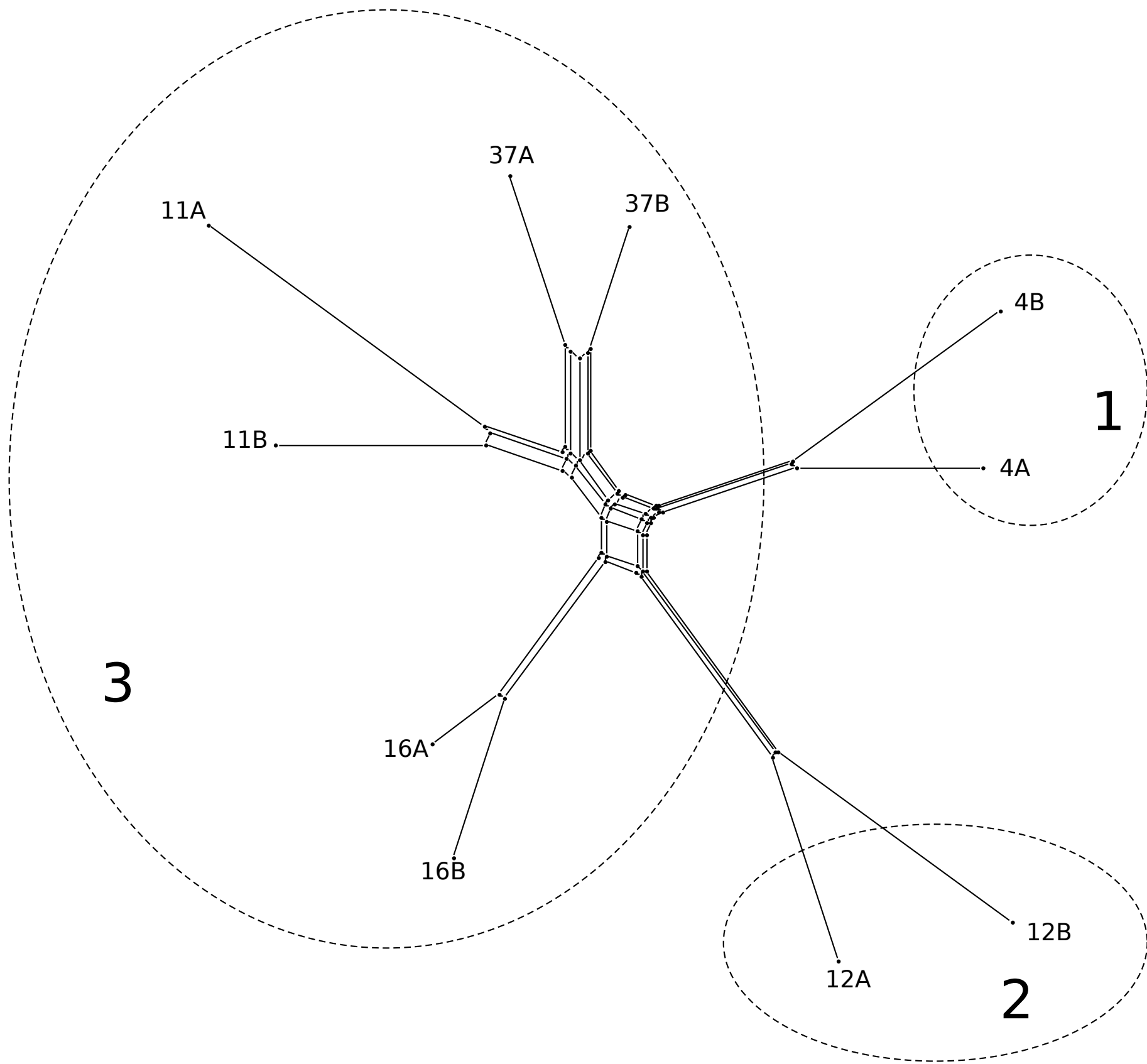

Supplement: Supplementary file 2 — Bayesian analysis of population structure (BAPS) clusters of paired meningococcal carriage isolates per sequence type. Neighbor-net splits trees of allelic differences in N. meningitidis core genome MLST genes (N. meningitidis cgMLST v1.0, available at pubMLST.org). Panel A, B, C and D show sequence types (ST) ST-11, ST-53, ST-192 and ST-2880, respectively. BAPS clusters are highlighted by circles and numbered sequentially. Paired isolates from the same individual that have been assigned to different BAPS clusters are underlined and connected with dashed lines. (ZIP 56 kb) [file 12864_2017_3806_MOESM2_ESM.zip › Suppl 1A cgMLST_st11_BAPS.pdf]

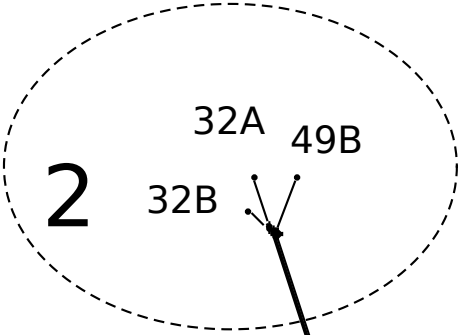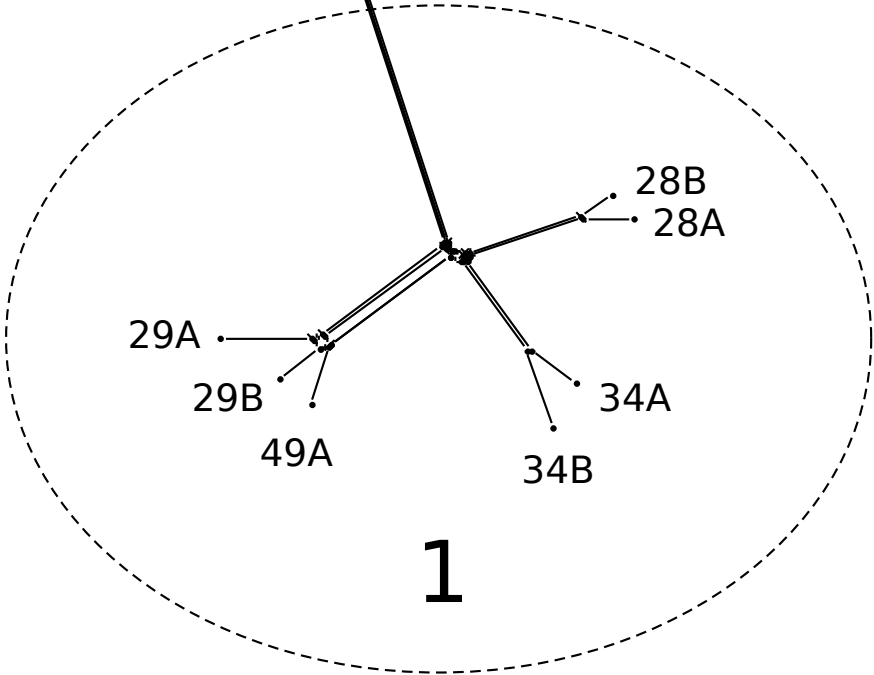

Supplement: Supplementary file 2 — Bayesian analysis of population structure (BAPS) clusters of paired meningococcal carriage isolates per sequence type. Neighbor-net splits trees of allelic differences in N. meningitidis core genome MLST genes (N. meningitidis cgMLST v1.0, available at pubMLST.org). Panel A, B, C and D show sequence types (ST) ST-11, ST-53, ST-192 and ST-2880, respectively. BAPS clusters are highlighted by circles and numbered sequentially. Paired isolates from the same individual that have been assigned to different BAPS clusters are underlined and connected with dashed lines. (ZIP 56 kb) [file 12864_2017_3806_MOESM2_ESM.zip › Suppl 1B cgMLST_st53_BAPS.pdf]

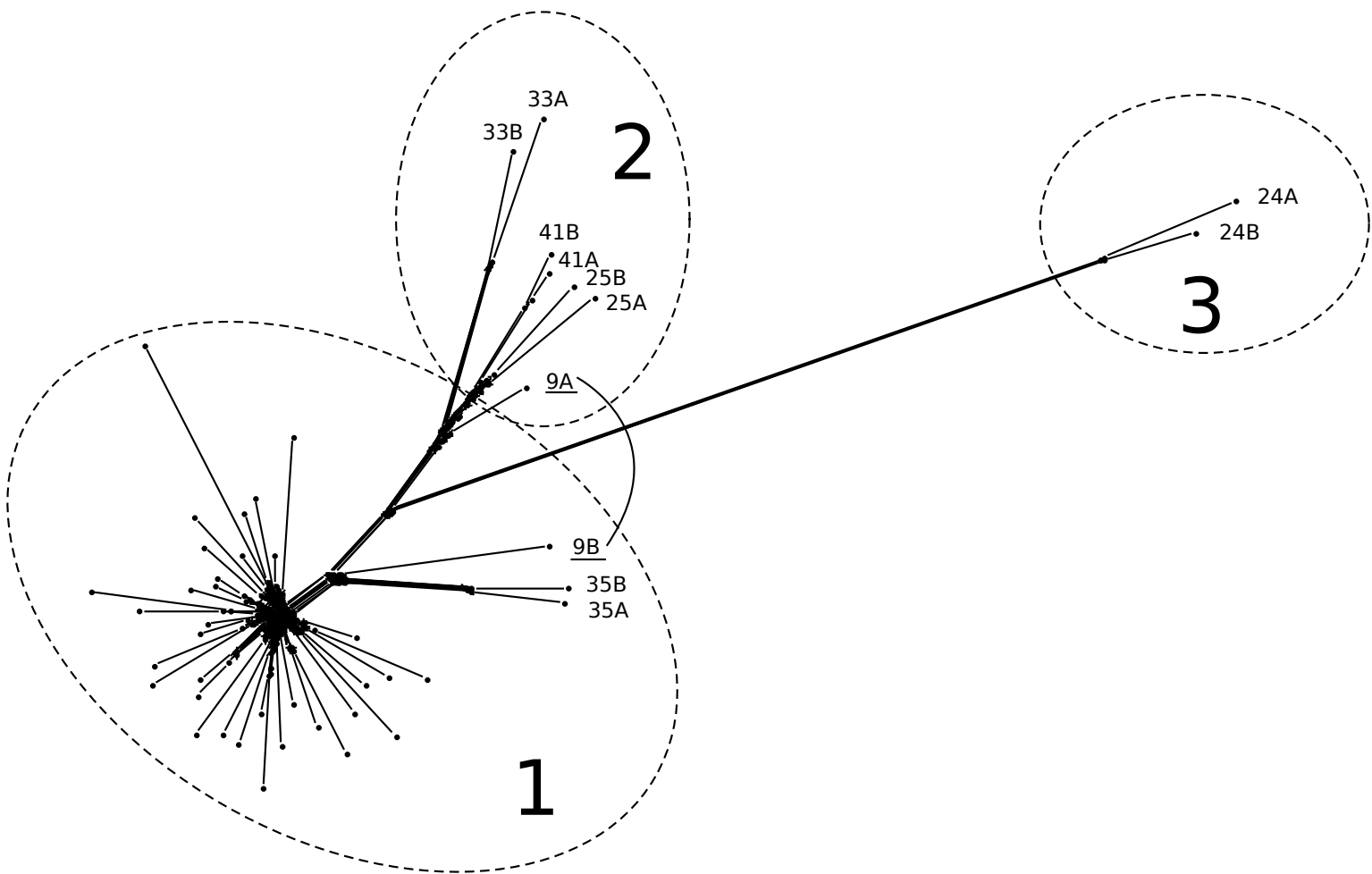

Supplement: Supplementary file 2 — Bayesian analysis of population structure (BAPS) clusters of paired meningococcal carriage isolates per sequence type. Neighbor-net splits trees of allelic differences in N. meningitidis core genome MLST genes (N. meningitidis cgMLST v1.0, available at pubMLST.org). Panel A, B, C and D show sequence types (ST) ST-11, ST-53, ST-192 and ST-2880, respectively. BAPS clusters are highlighted by circles and numbered sequentially. Paired isolates from the same individual that have been assigned to different BAPS clusters are underlined and connected with dashed lines. (ZIP 56 kb) [file 12864_2017_3806_MOESM2_ESM.zip › Suppl 1C cgMLST_st192_BAPS.pdf]

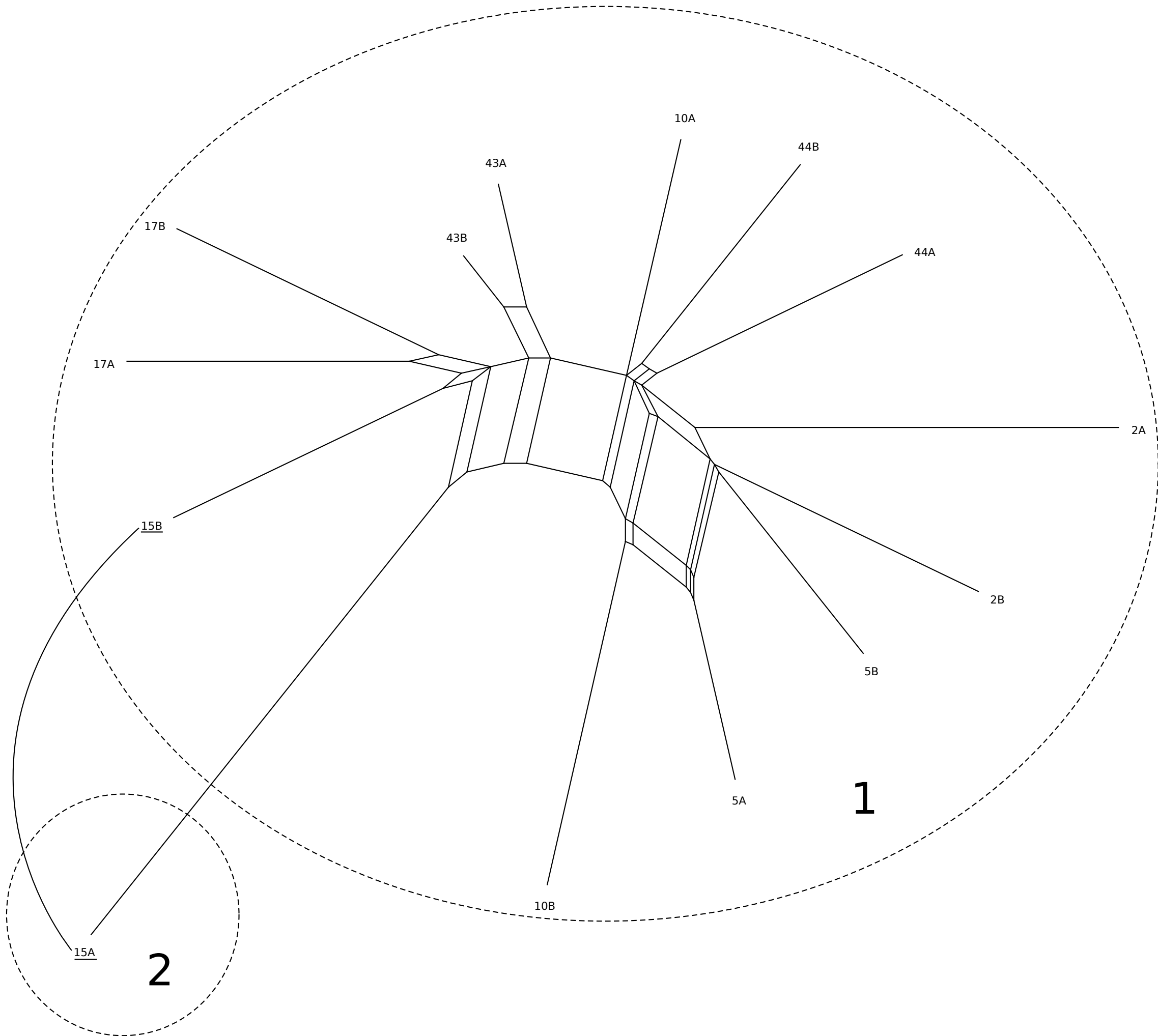

Supplement: Supplementary file 2 — Bayesian analysis of population structure (BAPS) clusters of paired meningococcal carriage isolates per sequence type. Neighbor-net splits trees of allelic differences in N. meningitidis core genome MLST genes (N. meningitidis cgMLST v1.0, available at pubMLST.org). Panel A, B, C and D show sequence types (ST) ST-11, ST-53, ST-192 and ST-2880, respectively. BAPS clusters are highlighted by circles and numbered sequentially. Paired isolates from the same individual that have been assigned to different BAPS clusters are underlined and connected with dashed lines. (ZIP 56 kb) [file 12864_2017_3806_MOESM2_ESM.zip › Suppl 1D cgMLST_st2880_BAPS.pdf]
