# Supplementary material for: Whole genome sequencing reveals within-host genetic changes in paired meningococcal carriage isolates from Ethiopia
Source: BMC Genomics. 2017 May 25;18:407. doi: 10.1186/s12864-017-3806-3 (PMC5445459; doi:10.1186/s12864-017-3806-3)
Supplement: Supplementary file 3 — Comparison of meningococcal carriage isolates in sequence types 11, 53, 192 and 2880. Number of allelic differences in the 1,605 genes of the N. meningitidis core genome*. (DOCX 55 kb) [file 12864_2017_3806_MOESM3_ESM.docx]

**Additional file 3: Table S2A**

**Comparison of meningococcal carriage isolates in sequence type 11**

Number of allelic differences in the 1,605 genes of the *N. meningitidis* core genome^*^.

| Isolate | 4A | 4B | 11A | 11B | 12A | 12B | 16A | 16B | 37A | 37B |
| --- | --- | --- | --- | --- | --- | --- | --- | --- | --- | --- |
| 4A | 0 |  |  |  |  |  |  |  |  |  |
| 4B | 37 | 0 |  |  |  |  |  |  |  |  |
| 11A | 72 | 78 | 0 |  |  |  |  |  |  |  |
| 11B | 62 | 68 | 47 | 0 |  |  |  |  |  |  |
| 12A | 68 | 76 | 83 | 71 | 0 |  |  |  |  |  |
| 12B | 73 | 78 | 88 | 74 | 40 | 0 |  |  |  |  |
| 16A | 58 | 63 | 67 | 54 | 60 | 65 | 0 |  |  |  |
| 16B | 64 | 68 | 73 | 62 | 67 | 72 | 21 | 0 |  |  |
| 37A | 54 | 66 | 58 | 49 | 74 | 77 | 53 | 55 | 0 |  |
| 37B | 54 | 59 | 57 | 46 | 70 | 75 | 48 | 62 | 28 | 0 |

^*^ 1,605 genes defined as the core genome (*N. meningitidis* cgMLST v1.0) in the database pubMLST.org.

**Supplementary table 2B**

**Comparison of meningococcal carriage isolates in sequence type 53**

Number of allelic differences in the 1,605 genes of the *N. meningitidis* core genome^*^.

| Isolate | 28A | 28B | 29A | 29B | 32A | 32B | 34A | 34B | 49A | 49B |
| --- | --- | --- | --- | --- | --- | --- | --- | --- | --- | --- |
| 28A | 0 |  |  |  |  |  |  |  |  |  |
| 28B | 32 | 0 |  |  |  |  |  |  |  |  |
| 29A | 162 | 154 | 0 |  |  |  |  |  |  |  |
| 29B | 143 | 133 | 55 | 0 |  |  |  |  |  |  |
| 32A | 468 | 466 | 488 | 479 | 0 |  |  |  |  |  |
| 32B | 458 | 457 | 481 | 471 | 27 | 0 |  |  |  |  |
| 34A | 119 | 115 | 154 | 133 | 469 | 460 | 0 |  |  |  |
| 34B | 127 | 113 | 162 | 145 | 477 | 466 | 48 | 0 |  |  |
| 49A | 141 | 134 | 62 | 42 | 479 | 471 | 137 | 146 | 0 |  |
| 49B | 467 | 463 | 489 | 480 | 42 | 34 | 471 | 474 | 473 | 0 |

^*^ 1,605 genes defined as the core genome (*N. meningitidis* cgMLST v1.0) in the database pubMLST.org.

**Supplementary table 2C**

**Comparison of meningococcal carriage isolates in sequence type 192**

Number of allelic differences in the 1,605 genes of the *N. meningitidis* core genome^*^.

| **Isolate** | **1A** | **1B** | **3A** | **3B** | **6A** | **6B** | **7A** | **7B** | **8A** | **8B** | **9A** | **9B** | **18A** | **18B** | **20A** | **20B** | **21A** | **21B** | **22A** | **22B** | **23A** | **23B** | **24A** | **24B** |
| --- | --- | --- | --- | --- | --- | --- | --- | --- | --- | --- | --- | --- | --- | --- | --- | --- | --- | --- | --- | --- | --- | --- | --- | --- |
| **1A** | 0 |  |  |  |  |  |  |  |  |  |  |  |  |  |  |  |  |  |  |  |  |  |  |  |
| **1B** | 34 | 0 |  |  |  |  |  |  |  |  |  |  |  |  |  |  |  |  |  |  |  |  |  |  |
| **3A** | 43 | 40 | 0 |  |  |  |  |  |  |  |  |  |  |  |  |  |  |  |  |  |  |  |  |  |
| **3B** | 39 | 41 | 42 | 0 |  |  |  |  |  |  |  |  |  |  |  |  |  |  |  |  |  |  |  |  |
| **6A** | 40 | 42 | 48 | 45 | 0 |  |  |  |  |  |  |  |  |  |  |  |  |  |  |  |  |  |  |  |
| **6B** | 27 | 32 | 37 | 33 | 28 | 0 |  |  |  |  |  |  |  |  |  |  |  |  |  |  |  |  |  |  |
| **7A** | 42 | 41 | 46 | 43 | 51 | 37 | 0 |  |  |  |  |  |  |  |  |  |  |  |  |  |  |  |  |  |
| **7B** | 40 | 43 | 46 | 41 | 50 | 35 | 38 | 0 |  |  |  |  |  |  |  |  |  |  |  |  |  |  |  |  |
| **8A** | 44 | 42 | 45 | 45 | 52 | 38 | 37 | 37 | 0 |  |  |  |  |  |  |  |  |  |  |  |  |  |  |  |
| **8B** | 37 | 34 | 33 | 37 | 44 | 29 | 27 | 29 | 26 | 0 |  |  |  |  |  |  |  |  |  |  |  |  |  |  |
| **9A** | 83 | 82 | 84 | 85 | 87 | 78 | 76 | 77 | 77 | 67 | 0 |  |  |  |  |  |  |  |  |  |  |  |  |  |
| **9B** | 69 | 69 | 74 | 76 | 78 | 70 | 64 | 70 | 74 | 61 | 84 | 0 |  |  |  |  |  |  |  |  |  |  |  |  |
| **18A** | 41 | 41 | 44 | 44 | 43 | 34 | 42 | 34 | 42 | 32 | 77 | 69 | 0 |  |  |  |  |  |  |  |  |  |  |  |
| **18B** | 53 | 53 | 50 | 57 | 61 | 47 | 49 | 44 | 46 | 38 | 87 | 72 | 45 | 0 |  |  |  |  |  |  |  |  |  |  |
| **20A** | 46 | 48 | 50 | 45 | 54 | 38 | 50 | 43 | 49 | 41 | 90 | 75 | 46 | 55 | 0 |  |  |  |  |  |  |  |  |  |
| **20B** | 40 | 39 | 42 | 38 | 46 | 31 | 40 | 38 | 42 | 34 | 81 | 73 | 42 | 51 | 36 | 0 |  |  |  |  |  |  |  |  |
| **21A** | 44 | 43 | 46 | 40 | 50 | 38 | 41 | 37 | 43 | 32 | 79 | 72 | 41 | 53 | 49 | 38 | 0 |  |  |  |  |  |  |  |
| **21B** | 52 | 51 | 55 | 50 | 57 | 47 | 47 | 43 | 45 | 37 | 83 | 76 | 45 | 55 | 52 | 44 | 41 | 0 |  |  |  |  |  |  |
| **22A** | 48 | 48 | 51 | 48 | 54 | 43 | 44 | 45 | 45 | 36 | 81 | 75 | 49 | 54 | 50 | 44 | 48 | 54 | 0 |  |  |  |  |  |
| **22B** | 45 | 43 | 47 | 48 | 50 | 40 | 41 | 43 | 43 | 34 | 81 | 69 | 44 | 55 | 48 | 41 | 47 | 53 | 38 | 0 |  |  |  |  |
| **23A** | 41 | 45 | 46 | 46 | 52 | 41 | 41 | 44 | 46 | 34 | 78 | 69 | 46 | 54 | 52 | 48 | 46 | 55 | 46 | 44 | 0 |  |  |  |
| **23B** | 56 | 58 | 58 | 54 | 59 | 45 | 53 | 53 | 55 | 46 | 89 | 78 | 53 | 65 | 56 | 51 | 53 | 60 | 54 | 51 | 36 | 0 |  |  |
| **24A** | 200 | 203 | 204 | 205 | 214 | 199 | 195 | 199 | 201 | 190 | 184 | 208 | 199 | 205 | 207 | 199 | 207 | 205 | 201 | 203 | 204 | 207 | 0 |  |
| **24B** | 196 | 190 | 195 | 199 | 199 | 191 | 189 | 193 | 194 | 183 | 180 | 197 | 189 | 197 | 197 | 191 | 199 | 200 | 196 | 197 | 196 | 201 | 40 | 0 |
| **25A** | 101 | 105 | 150 | 102 | 109 | 94 | 99 | 92 | 100 | 86 | 51 | 98 | 97 | 99 | 103 | 97 | 98 | 101 | 101 | 98 | 99 | 103 | 208 | 199 |
| **25B** | 97 | 99 | 102 | 101 | 109 | 94 | 95 | 97 | 96 | 84 | 49 | 102 | 97 | 103 | 107 | 97 | 98 | 106 | 98 | 100 | 98 | 108 | 204 | 196 |
| **31A** | 54 | 50 | 54 | 49 | 57 | 46 | 50 | 40 | 48 | 42 | 88 | 80 | 43 | 58 | 52 | 47 | 49 | 52 | 58 | 53 | 54 | 61 | 203 | 195 |
| **31B** | 44 | 38 | 43 | 41 | 52 | 37 | 36 | 39 | 33 | 27 | 73 | 64 | 41 | 44 | 45 | 35 | 38 | 45 | 40 | 40 | 41 | 50 | 194 | 185 |
| **33A** | 118 | 119 | 120 | 119 | 126 | 110 | 119 | 120 | 114 | 108 | 78 | 131 | 121 | 126 | 124 | 118 | 119 | 127 | 122 | 122 | 124 | 132 | 224 | 218 |
| **33B** | 114 | 117 | 119 | 110 | 120 | 107 | 113 | 114 | 114 | 103 | 71 | 122 | 112 | 122 | 119 | 109 | 110 | 117 | 113 | 115 | 115 | 119 | 220 | 213 |
| **35A** | 75 | 76 | 77 | 70 | 81 | 69 | 73 | 69 | 75 | 63 | 87 | 78 | 71 | 75 | 74 | 70 | 68 | 78 | 75 | 75 | 74 | 81 | 213 | 202 |
| **35B** | 75 | 71 | 76 | 74 | 83 | 69 | 71 | 69 | 72 | 61 | 88 | 75 | 70 | 79 | 77 | 74 | 72 | 84 | 78 | 76 | 72 | 83 | 213 | 200 |
| **36A** | 39 | 35 | 40 | 41 | 45 | 32 | 33 | 39 | 41 | 27 | 75 | 64 | 41 | 48 | 45 | 37 | 37 | 48 | 40 | 37 | 39 | 50 | 201 | 189 |
| **36B** | 41 | 38 | 39 | 41 | 46 | 34 | 39 | 41 | 41 | 28 | 74 | 68 | 45 | 52 | 48 | 37 | 40 | 47 | 44 | 41 | 40 | 52 | 200 | 192 |
| **38A** | 37 | 38 | 39 | 33 | 42 | 31 | 31 | 29 | 32 | 24 | 67 | 62 | 30 | 39 | 39 | 30 | 31 | 35 | 41 | 39 | 41 | 46 | 192 | 183 |
| **38B** | 35 | 33 | 37 | 36 | 42 | 29 | 24 | 26 | 27 | 16 | 63 | 55 | 29 | 35 | 38 | 30 | 28 | 35 | 32 | 30 | 33 | 43 | 188 | 181 |
| **39A** | 32 | 35 | 36 | 33 | 36 | 25 | 29 | 30 | 35 | 23 | 70 | 62 | 34 | 46 | 38 | 31 | 32 | 42 | 30 | 29 | 33 | 40 | 197 | 187 |
| **39B** | 33 | 35 | 38 | 37 | 42 | 31 | 31 | 35 | 39 | 25 | 72 | 56 | 39 | 45 | 40 | 34 | 37 | 44 | 33 | 28 | 31 | 42 | 196 | 187 |
| **40A** | 39 | 40 | 41 | 43 | 46 | 35 | 31 | 37 | 38 | 21 | 75 | 57 | 39 | 45 | 47 | 39 | 38 | 44 | 41 | 39 | 39 | 51 | 202 | 189 |
| **40B** | 77 | 74 | 75 | 77 | 83 | 69 | 65 | 70 | 70 | 55 | 106 | 100 | 70 | 80 | 82 | 71 | 71 | 78 | 70 | 72 | 75 | 87 | 230 | 220 |
| **41A** | 99 | 97 | 101 | 97 | 104 | 91 | 93 | 91 | 98 | 84 | 50 | 101 | 96 | 104 | 103 | 95 | 94 | 107 | 97 | 97 | 98 | 107 | 206 | 196 |
| **41B** | 102 | 100 | 105 | 102 | 107 | 95 | 96 | 100 | 100 | 88 | 55 | 106 | 100 | 108 | 107 | 98 | 97 | 111 | 99 | 100 | 101 | 109 | 205 | 198 |
| **45A** | 36 | 30 | 36 | 35 | 42 | 28 | 30 | 34 | 31 | 21 | 69 | 61 | 37 | 43 | 40 | 30 | 31 | 42 | 35 | 32 | 36 | 44 | 193 | 185 |
| **45B** | 36 | 31 | 38 | 34 | 44 | 30 | 30 | 34 | 30 | 22 | 69 | 62 | 37 | 45 | 38 | 31 | 33 | 41 | 36 | 32 | 33 | 42 | 198 | 185 |
| **46A** | 30 | 36 | 39 | 34 | 40 | 26 | 34 | 31 | 35 | 28 | 74 | 66 | 37 | 47 | 39 | 28 | 34 | 41 | 41 | 37 | 42 | 46 | 196 | 188 |
| **46B** | 40 | 35 | 36 | 36 | 48 | 33 | 36 | 38 | 38 | 26 | 74 | 59 | 40 | 48 | 41 | 35 | 34 | 46 | 37 | 35 | 34 | 45 | 199 | 189 |

| **Isolate** | **25A** | **25B** | **31A** | **31B** | **33A** | **33B** | **35A** | **35B** | **36A** | **36B** | **38A** | **38B** | **39A** | **39B** | **40A** | **40B** | **41A** | **41B** | **45A** | **45B** | **46A** | **46B** |
| --- | --- | --- | --- | --- | --- | --- | --- | --- | --- | --- | --- | --- | --- | --- | --- | --- | --- | --- | --- | --- | --- | --- |
| **1A** |  |  |  |  |  |  |  |  |  |  |  |  |  |  |  |  |  |  |  |  |  |  |
| **1B** |  |  |  |  |  |  |  |  |  |  |  |  |  |  |  |  |  |  |  |  |  |  |
| **3A** |  |  |  |  |  |  |  |  |  |  |  |  |  |  |  |  |  |  |  |  |  |  |
| **3B** |  |  |  |  |  |  |  |  |  |  |  |  |  |  |  |  |  |  |  |  |  |  |
| **6A** |  |  |  |  |  |  |  |  |  |  |  |  |  |  |  |  |  |  |  |  |  |  |
| **6B** |  |  |  |  |  |  |  |  |  |  |  |  |  |  |  |  |  |  |  |  |  |  |
| **7A** |  |  |  |  |  |  |  |  |  |  |  |  |  |  |  |  |  |  |  |  |  |  |
| **7B** |  |  |  |  |  |  |  |  |  |  |  |  |  |  |  |  |  |  |  |  |  |  |
| **8A** |  |  |  |  |  |  |  |  |  |  |  |  |  |  |  |  |  |  |  |  |  |  |
| **8B** |  |  |  |  |  |  |  |  |  |  |  |  |  |  |  |  |  |  |  |  |  |  |
| **9A** |  |  |  |  |  |  |  |  |  |  |  |  |  |  |  |  |  |  |  |  |  |  |
| **9B** |  |  |  |  |  |  |  |  |  |  |  |  |  |  |  |  |  |  |  |  |  |  |
| **18A** |  |  |  |  |  |  |  |  |  |  |  |  |  |  |  |  |  |  |  |  |  |  |
| **18B** |  |  |  |  |  |  |  |  |  |  |  |  |  |  |  |  |  |  |  |  |  |  |
| **20A** |  |  |  |  |  |  |  |  |  |  |  |  |  |  |  |  |  |  |  |  |  |  |
| **20B** |  |  |  |  |  |  |  |  |  |  |  |  |  |  |  |  |  |  |  |  |  |  |
| **21A** |  |  |  |  |  |  |  |  |  |  |  |  |  |  |  |  |  |  |  |  |  |  |
| **21B** |  |  |  |  |  |  |  |  |  |  |  |  |  |  |  |  |  |  |  |  |  |  |
| **22A** |  |  |  |  |  |  |  |  |  |  |  |  |  |  |  |  |  |  |  |  |  |  |
| **22B** |  |  |  |  |  |  |  |  |  |  |  |  |  |  |  |  |  |  |  |  |  |  |
| **23A** |  |  |  |  |  |  |  |  |  |  |  |  |  |  |  |  |  |  |  |  |  |  |
| **23B** |  |  |  |  |  |  |  |  |  |  |  |  |  |  |  |  |  |  |  |  |  |  |
| **24A** |  |  |  |  |  |  |  |  |  |  |  |  |  |  |  |  |  |  |  |  |  |  |
| **24B** |  |  |  |  |  |  |  |  |  |  |  |  |  |  |  |  |  |  |  |  |  |  |
| **25A** | 0 |  |  |  |  |  |  |  |  |  |  |  |  |  |  |  |  |  |  |  |  |  |
| **25B** | 44 | 0 |  |  |  |  |  |  |  |  |  |  |  |  |  |  |  |  |  |  |  |  |
| **31A** | 108 | 106 | 0 |  |  |  |  |  |  |  |  |  |  |  |  |  |  |  |  |  |  |  |
| **31B** | 92 | 89 | 40 | 0 |  |  |  |  |  |  |  |  |  |  |  |  |  |  |  |  |  |  |
| **33A** | 88 | 84 | 129 | 114 | 0 |  |  |  |  |  |  |  |  |  |  |  |  |  |  |  |  |  |
| **33B** | 80 | 80 | 123 | 109 | 46 | 0 |  |  |  |  |  |  |  |  |  |  |  |  |  |  |  |  |
| **35A** | 103 | 103 | 76 | 62 | 130 | 117 | 0 |  |  |  |  |  |  |  |  |  |  |  |  |  |  |  |
| **35B** | 108 | 104 | 76 | 67 | 127 | 119 | 36 | 0 |  |  |  |  |  |  |  |  |  |  |  |  |  |  |
| **36A** | 98 | 93 | 48 | 33 | 116 | 109 | 69 | 69 | 0 |  |  |  |  |  |  |  |  |  |  |  |  |  |
| **36B** | 100 | 94 | 52 | 37 | 118 | 108 | 70 | 71 | 16 | 0 |  |  |  |  |  |  |  |  |  |  |  |  |
| **38A** | 89 | 92 | 41 | 31 | 116 | 102 | 59 | 64 | 34 | 31 | 0 |  |  |  |  |  |  |  |  |  |  |  |
| **38B** | 84 | 85 | 39 | 24 | 110 | 102 | 57 | 58 | 26 | 28 | 20 | 0 |  |  |  |  |  |  |  |  |  |  |
| **39A** | 90 | 88 | 43 | 31 | 112 | 100 | 62 | 62 | 26 | 30 | 26 | 22 | 0 |  |  |  |  |  |  |  |  |  |
| **39B** | 91 | 90 | 49 | 31 | 113 | 104 | 66 | 67 | 26 | 30 | 30 | 22 | 19 | 0 |  |  |  |  |  |  |  |  |
| **40A** | 93 | 92 | 50 | 32 | 119 | 111 | 69 | 70 | 31 | 34 | 30 | 21 | 30 | 26 | 0 |  |  |  |  |  |  |  |
| **40B** | 127 | 121 | 83 | 65 | 147 | 142 | 101 | 101 | 65 | 72 | 65 | 59 | 62 | 66 | 60 | 0 |  |  |  |  |  |  |
| **41A** | 52 | 44 | 106 | 90 | 85 | 81 | 107 | 103 | 91 | 94 | 90 | 83 | 85 | 86 | 92 | 123 | 0 |  |  |  |  |  |
| **41B** | 57 | 52 | 107 | 91 | 88 | 85 | 107 | 106 | 93 | 96 | 93 | 86 | 89 | 89 | 95 | 126 | 15 | 0 |  |  |  |  |
| **45A** | 89 | 85 | 46 | 24 | 108 | 105 | 64 | 65 | 26 | 28 | 28 | 19 | 21 | 23 | 30 | 62 | 83 | 87 | 0 |  |  |  |
| **45B** | 90 | 86 | 42 | 26 | 108 | 105 | 66 | 67 | 28 | 32 | 28 | 23 | 23 | 25 | 32 | 63 | 85 | 89 | 14 | 0 |  |  |
| **46A** | 90 | 93 | 43 | 32 | 112 | 106 | 69 | 67 | 32 | 34 | 25 | 24 | 24 | 28 | 35 | 69 | 92 | 93 | 25 | 27 | 0 |  |
| **46B** | 92 | 89 | 44 | 27 | 114 | 109 | 67 | 66 | 30 | 33 | 31 | 25 | 27 | 26 | 32 | 64 | 88 | 91 | 22 | 22 | 29 | 0 |

^*^ 1,605 genes defined as the core genome (*N. meningitidis* cgMLST v1.0) in the database pubMLST.org.

**Supplementary table 2D**

**Comparison of meningococcal carriage isolates in sequence type 2880**

Number of allelic differences in the 1,605 genes of the *N. meningitidis* core genome^*^.

| Isolate | 2A | 2B | 5A | 5B | 10A | 10B | 15A | 15B | 17A | 17B | 43A | 43B | 44A | 44B |
| --- | --- | --- | --- | --- | --- | --- | --- | --- | --- | --- | --- | --- | --- | --- |
| 2A | 0 |  |  |  |  |  |  |  |  |  |  |  |  |  |
| 2B | 40 | 0 |  |  |  |  |  |  |  |  |  |  |  |  |
| 5A | 38 | 31 | 0 |  |  |  |  |  |  |  |  |  |  |  |
| 5B | 38 | 28 | 28 | 0 |  |  |  |  |  |  |  |  |  |  |
| 10A | 39 | 33 | 44 | 28 | 0 |  |  |  |  |  |  |  |  |  |
| 10B | 52 | 43 | 32 | 43 | 33 | 0 |  |  |  |  |  |  |  |  |
| 15A | 68 | 68 | 46 | 62 | 56 | 69 | 0 |  |  |  |  |  |  |  |
| 15B | 49 | 45 | 65 | 43 | 37 | 51 | 52 | 0 |  |  |  |  |  |  |
| 17A | 52 | 48 | 48 | 46 | 38 | 54 | 56 | 34 | 0 |  |  |  |  |  |
| 17B | 48 | 46 | 48 | 45 | 37 | 52 | 53 | 36 | 31 | 0 |  |  |  |  |
| 43A | 41 | 36 | 33 | 31 | 27 | 40 | 48 | 29 | 32 | 32 | 0 |  |  |  |
| 43B | 39 | 32 | 30 | 32 | 25 | 37 | 44 | 26 | 27 | 27 | 11 | 0 |  |  |
| 44A | 43 | 36 | 37 | 30 | 29 | 42 | 60 | 37 | 41 | 41 | 31 | 29 | 0 |  |
| 44B | 42 | 36 | 35 | 35 | 27 | 41 | 57 | 37 | 39 | 39 | 28 | 27 | 28 | 0 |

^*^ 1,605 genes defined as the core genome (*N. meningitidis* cgMLST v1.0) in the database pubMLST.org
