# Supplementary material for: Whole genome sequencing reveals within-host genetic changes in paired meningococcal carriage isolates from Ethiopia
Source: BMC Genomics. 2017 May 25;18:407. doi: 10.1186/s12864-017-3806-3 (PMC5445459; doi:10.1186/s12864-017-3806-3)
Supplement: Supplementary file 4 — Mechanism of genetic change in paired meningococcal carriage isolates in sequence types 11, 53, 192 and 2880. (DOCX 59 kb) [file 12864_2017_3806_MOESM4_ESM.docx]

**Additional file 4: Table S3A**

**Mechanism of genetic change in paired meningococcal carriage isolates in sequence type 11**

| **Main mechanism of change in all STs** | **Gene conversion** | **Mutation**  **Recombi-nation** | **Gene conversion** | **Phase variation (AGCC)** | **Phase variation (CTTCT)** | **Phase variation (CTTCT)** | **Phase variation (polyC)** | **Mutation** | **Phase variation (polyG)** | **Phase variation (polyG)** | **Recombi-nation** | **Phase variation (polyG)** | **Phase variation (polyG)** | **Phase variation (polyG)** | **Recombi-nation** | **Recombi-nation**  **Deletion** | **Mutation** |
| --- | --- | --- | --- | --- | --- | --- | --- | --- | --- | --- | --- | --- | --- | --- | --- | --- | --- |
| **Gene** | pilE | unknown | pilS | modA | opaB | opa1800 | pglG | relA | pglH | pglI | tspA | lgtA | hypotetical | hpuA | putative | putative | emrB |
| NEIS no | NEIS0210 | NEIS2649 | pilS | NEIS1310 | NEIS1403 | NEIS1719 | NEIS0401 | NEIS1655 | NEIS0400 | NEIS0380 | NEIS1829 | NEIS1902 | NEIS1750 | NEIS1946 | NEIS1288 | NEIS1418 | NEIS1852 |
| **Individual** |  |  |  |  |  |  |  |  |  |  |  |  |  |  |  |  |  |
| **4** | - | PM* | - | PV | PV | PV | I | PM | - | PV | - | - | - | - | - | - | PM* |
|  |  |  |  |  |  |  |  |  |  |  |  |  |  |  |  |  |  |
| **11** | - | PM | - | PV | - | - | - | - | PV | PV | - | - | - | PV | R | - | - |
|  |  |  |  |  |  |  |  |  |  |  |  |  |  |  |  |  |  |
| **12** | - | PM* | - | PV | - | - | PV | - | PV | PV | - | - | - | - | PM | - | PM* |
|  |  |  |  |  |  |  |  |  |  |  |  |  |  |  |  |  |  |
| **16** | - | PM* | - | PV | - | - | - | - | PV | - | - | - | PV | PV | - | PM | - |
|  |  |  |  |  |  |  |  |  |  |  |  |  |  |  |  |  |  |
| **37** | - | R* | - | PV | - | - | PV | PM* | - | - | - | - | - | - | - | - | PM* |
|  |  |  |  |  |  |  |  |  |  |  |  |  |  |  |  |  |  |

^*^PM or R seen in the same position(s) in the gene in more than one pair.

PM: point mutation, PV: phase variation, R: recombination, I: incomplete sequence

**Supplementary table 3B**

**Mechanism of genetic change in paired meningococcal carriage isolates in sequence type 53**

| **Main mechanism of change in all STs** | **Gene conversion** | **Mutation**  **Recombi-nation** | **Gene conversion** | **Phase variation (AGCC)** | **Phase variation (CTTCT)** | **Phase variation (CTTCT)** | **Phase variation (polyC)** | **Mutation** | **Phase variation (polyG)** | **Phase variation (polyG)** | **Recombi-nation** | **Phase variation (polyG)** | **Phase variation (polyG)** | **Phase variation (polyG)** | **Recombi-nation** | **Recombi-nation**  **Deletion** | **Mutation** |
| --- | --- | --- | --- | --- | --- | --- | --- | --- | --- | --- | --- | --- | --- | --- | --- | --- | --- |
| **Gene** | pilE | unknown | pilS | modA | opaB | opa1800 | pglG | relA | pglH | pglI | tspA | lgtA | hypotetical | hpuA | putative | putative | emrB |
| NEIS no | NEIS0210 | NEIS2649 | pilS | NEIS1310 | NEIS1403 | NEIS1719 | NEIS0401 | NEIS1655 | NEIS0400 | NEIS0380 | NEIS1829 | NEIS1902 | NEIS1750 | NEIS1946 | NEIS1288 | NEIS1418 | NEIS1852 |
| **Individual** |  |  |  |  |  |  |  |  |  |  |  |  |  |  |  |  |  |
| **28** | GC | R* | - | - | PV | PV | - | - | - | - | R | - | - | - | PM* | PM | PM* |
|  |  |  |  |  |  |  |  |  |  |  |  |  |  |  |  |  |  |
| **29** | - | - | GC | - | PV | PV | PV | - | - | - | - | - | - | - | - | - | R* |
|  |  |  |  |  |  |  |  |  |  |  |  |  |  |  |  |  |  |
| **32** | GC | - | GC | - | - | - | PV | - | - | - | - | - | - | - | PM* | - | - |
|  |  |  |  |  |  |  |  |  |  |  |  |  |  |  |  |  |  |
| **34** | GC | - | GC | - | - | - | I | PM* | - | - | D | - | - | - | PM* | D | PM* |
|  |  |  |  |  |  |  |  |  |  |  |  |  |  |  |  |  |  |

^*^PM seen in the same position(s) in the gene in more than one pair.

D: Deletion, GC: gene conversion, PM: point mutation, PV: phase variation, R: recombination, I: incomplete sequence

**Supplementary table 3C**

**Mechanism of genetic change in paired meningococcal carriage isolates in sequence type 192**

| **Main mechanism of change in all STs** | **Gene conversion** | **Mutation**  **Recombi-nation** | **Gene conversion** | **Phase variation (AGCC)** | **Phase variation (CTTCT)** | **Phase variation (CTTCT)** | **Phase variation (polyC)** | **Mutation** | **Phase variation (polyG)** | **Phase variation (polyG)** | **Recombi-nation** | **Phase variation (polyG)** | **Phase variation (polyG)** | **Phase variation (polyG)** | **Recombi-nation** | **Recombi-nation**  **Deletion** | **Mutation** |
| --- | --- | --- | --- | --- | --- | --- | --- | --- | --- | --- | --- | --- | --- | --- | --- | --- | --- |
| **Gene** | pilE | unknown | pilS | modA | opaB | opa1800 | pglG | relA | pglH | pglI | tspA | lgtA | hypotetical | hpuA | putative | putative | emrB |
| NEIS no | NEIS0210 | NEIS2649 | pilS | NEIS1310 | NEIS1403 | NEIS1719 | NEIS0401 | NEIS1655 | NEIS0400 | NEIS0380 | NEIS1829 | NEIS1902 | NEIS1750 | NEIS1946 | NEIS1288 | NEIS1418 | NEIS1852 |
| **Individual** |  |  |  |  |  |  |  |  |  |  |  |  |  |  |  |  |  |
| **1** | GC | PM* | GC | PV | PV | PV | PV | - | PV | - | R* | PV | - | - | - | - | - |
|  |  |  |  |  |  |  |  |  |  |  |  |  |  |  |  |  |  |
| **3** | GC | - | GC | PV | - | - | PV | - | PV | - | D | PV | PV | - | R | - | - |
|  |  |  |  |  |  |  |  |  |  |  |  |  |  |  |  |  |  |
| **6** | GC | - | GC | PV | PV | PV | PV | - | PV | - | - | PV | - | PV | - | - | - |
|  |  |  |  |  |  |  |  |  |  |  |  |  |  |  |  |  |  |
| **7** | GC | - | GC | - | - | - | PV | - | PV | PV | - | PV | - | PV | - | PM* | - |
|  |  |  |  |  |  |  | R |  |  |  |  |  |  |  |  |  |  |
| **8** | GC | R | GC | PV | PV | PV | PV | - | - | PV | R* | PV | - | PV | - | D | - |
|  |  |  |  |  |  |  |  |  |  |  | D |  |  |  |  |  |  |
| **9** | GC | PM* | GC | PV | PV | PV | PV | PM* | PV | PV | - | PV | PM* | PV | - | - | - |
|  |  |  |  |  |  |  |  |  |  |  |  |  |  | PM |  |  |  |
| **18** | GC | R | GC | PV | PV | PV | PV | PM* | - | PV | R* | I | - | - | - | D | - |
|  |  |  |  |  |  |  |  |  |  |  |  |  |  |  |  |  |  |
| **20** | GC | R | GC | PV | PV | PV | - | - | PV | PV | R* | - | - | R | R | - | - |
|  |  |  |  |  |  |  |  |  |  |  | PM |  |  |  |  |  |  |
| **21** | GC | PM* | GC | PV | PV | PV | PV | PM* | PV | PV | R* | PV | PV | - | - | D | - |
|  |  |  |  |  |  |  |  |  |  |  |  | D |  |  |  | R |  |
| **22** | GC | - | GC | PV | PV | PV | - | - | PV | - | R* | - | PV | - | - | - | - |
|  |  |  |  |  |  |  |  |  |  |  |  |  |  |  |  |  |  |
| **23** | GC | - | GC | PV | PV | PV | - | PM* | PV | PV | R* | PV | - | PV | - | - | - |
|  |  |  |  |  |  |  |  |  |  |  | R |  |  |  |  |  |  |
| **24** | GC | PM* | - | - | PV | PV | - | R | PV | - | R* | PV | - | PV | R | D | PM* |
|  |  |  |  |  |  |  |  |  |  |  | D |  |  |  |  |  |  |
| **25** | GC | PM* | GC | PV | PV | PV | - | PM* | - | PV | D | I | PV | PV | - | - | - |
|  |  |  |  |  |  |  |  |  |  |  |  |  |  |  |  |  |  |
| **31** | GC | PM* | GC | PV | PV | PV | PV | PM* | - | PV | R | I | PV | PV | R | - | - |
|  |  |  |  |  |  |  |  |  |  |  | D | D |  |  |  |  |  |
| **33** | GC | R | GC | I | PV | PV | - | PM* | PV | - | R | PV | - | - | R | - | - |
|  |  |  |  |  |  |  |  |  |  |  |  |  |  |  |  |  |  |
| **35** | GC | PM* | GC | PV | - | - | PV | PM* | - | PV | - | I | PV | PV | R | - | - |
|  |  |  |  |  |  |  |  |  |  |  |  |  |  |  |  |  |  |
| **36** | GC | R | GC | PV | PV | PV | PV | - | PV | - | - | PV | - | PV | - | - | - |
|  |  |  |  |  |  |  |  |  |  |  |  |  |  |  |  |  |  |
| **38** | GC | PM* | GC | - | PV | PV | PV | - | - | PV | - | PV | - | PV | - | - | - |
|  |  |  |  |  |  | R |  |  |  |  |  |  |  |  |  |  |  |
| **39** | GC | R | GC | PV | - | - | - | PM* | PV | - | - | PV | - | - | - | - | - |
|  |  |  |  |  |  |  |  |  |  |  |  |  |  |  |  |  |  |
| **40** | GC | R | GC | PV | PV | PV | I | PM* | PV | - | R* | PV | PV | - | - | D | PM* |
|  |  |  |  |  |  |  |  |  |  |  |  |  |  |  |  | PM |  |
| **41** | - | R | GC | PV | - | - | - | - | PV | - | - | - | PV | - | - | - | - |
|  |  |  |  |  |  |  |  |  |  |  |  |  |  |  |  |  |  |
| **45** | GC | PM* | GC | PV | PV | PV | PV | - | PV | - | R* | I | PV | PV | - | - | - |
|  |  |  |  |  |  |  |  |  |  |  |  |  |  |  |  |  |  |
| **46** | GC | R | GC | PV | PV | PV | I | - | PV | PV | R* | - | PV | - | - | - | - |
|  |  |  |  |  |  | R | R |  |  |  |  |  |  |  |  |  |  |

^*^PM or R seen in the same position(s) in the gene in more than one pair.

D: Deletion, GC: gene conversion, PM: point mutation, PV: phase variation, R: recombination, I: incomplete sequence

**Supplementary table 3D**

**Mechanism of genetic change in paired meningococcal carriage isolates in sequence type 2880**

| **Main mechanism of change in all STs** | **Gene conversion** | **Mutation**  **Recombi-nation** | **Gene conversion** | **Phase variation (AGCC)** | **Phase variation (CTTCT)** | **Phase variation (CTTCT)** | **Phase variation (polyC)** | **Mutation** | **Phase variation (polyG)** | **Phase variation (polyG)** | **Recombi-nation** | **Phase variation (polyG)** | **Phase variation (polyG)** | **Phase variation (polyG)** | **Recombi-nation** | **Recombi-nation**  **Deletion** | **Mutation** |
| --- | --- | --- | --- | --- | --- | --- | --- | --- | --- | --- | --- | --- | --- | --- | --- | --- | --- |
| **Gene** | pilE | unknown | pilS | modA | opaB | opa1800 | pglG | relA | pglH | pglI | tspA | lgtA | hypotetical | hpuA | putative | putative | emrB |
| NEIS no | NEIS0210 | NEIS2649 | pilS | NEIS1310 | NEIS1403 | NEIS1719 | NEIS0401 | NEIS1655 | NEIS0400 | NEIS0380 | NEIS1829 | NEIS1902 | NEIS1750 | NEIS1946 | NEIS1288 | NEIS1418 | NEIS1852 |
| **Individual** |  |  |  |  |  |  |  |  |  |  |  |  |  |  |  |  |  |
| **2** | GC | R* | GC | - | - | - | - | - | - | PV | - | - | - | - | R* | R | PM* |
|  |  |  |  |  |  |  |  |  |  |  |  |  |  |  |  |  |  |
| **5** | GC | R* | - | PV | PV | PV | - | - | - | PV | - | - | - | - | PM* | - | PM* |
|  |  |  |  |  |  |  |  |  |  |  |  |  |  |  |  |  |  |
| **10** | GC | R* | - | PV | PV | PV | - | - | - | PV | - | - | - | - | R* | - | - |
|  |  |  |  |  |  |  |  |  |  |  |  |  |  |  |  |  |  |
| **15** | GC | PM* | GC | PV | - | - | - | PM* | PV | - | - | - | PV | - | - | - | R* |
|  |  |  |  |  |  |  |  |  |  |  |  |  |  |  |  |  |  |
| **17** | GC | R* | GC | - | - | - | - | PM* | - | - | - | - | PV | - | - | R | R* |
|  |  |  |  |  |  |  |  |  |  |  |  |  |  |  |  |  |  |
| **43** | GC | - | GC | PV | - | - | - | PM* | - | - | - | - | - | - | - | R | - |
|  |  |  |  |  |  |  |  |  |  |  |  |  |  |  |  |  |  |
| **44** | GC | - | GC | PV | - | - | - | PM* | - | - | - | - | - | - | PM | R | - |
|  |  |  |  |  |  |  |  |  |  |  |  |  |  |  |  |  |  |

^*^PM or R seen in the same position(s) in the gene in more than one pair.

GC: gene conversion, PM: point mutation, PV: phase variation, R: recombination
